# Selection and validation of reference genes for gene expression studies in *Klebsiella pneumoniae* using Reverse Transcription Quantitative real-time PCR

**DOI:** 10.1038/s41598-018-27420-2

**Published:** 2018-06-13

**Authors:** Ana Érika Inácio Gomes, Leonardo Prado Stuchi, Nathália Maria Gonçalves Siqueira, João Batista Henrique, Renato Vicentini, Marcelo Lima Ribeiro, Michelle Darrieux, Lúcio Fábio Caldas Ferraz

**Affiliations:** 1 Laboratório de Biologia Molecular de Microrganismos, Universidade São Francisco, Bragança Paulista, SP CEP 12916-900 Brazil; 2 Centro de Biologia Molecular e Engenharia Genética, Universidade Estadual de Campinas, Campinas, SP CEP 13083-875 Brazil; 3 Unidade Integrada de Farmacologia e Gastroenterologia (UNIFAG), Universidade São Francisco, Bragança Paulista, SP CEP 12916-900 Brazil

## Abstract

For reliable results, Reverse Transcription Quantitative real-time Polymerase Chain Reaction (RT-qPCR) analyses depend on stably expressed reference genes for data normalization purposes. *Klebsiella pneumoniae* is an opportunistic Gram-negative bacterium that has become a serious threat worldwide. Unfortunately, there is no consensus for an ideal reference gene for RT-qPCR data normalization on *K. pneumoniae*. In this study, the expression profile of eleven candidate reference genes was assessed in *K. pneumoniae* cells submitted to various experimental conditions, and the expression stability of these candidate genes was evaluated using statistical algorithms BestKeeper, NormFinder, geNorm, Delta C_T_ and RefFinder. The statistical analyses ranked *recA*, *rho*, *proC* and *rpoD* as the most suitable reference genes for accurate RT-qPCR data normalization in *K. pneumoniae*. The reliability of the proposed reference genes was validated by normalizing the relative expression of iron-regulated genes in *K. pneumoniae* cells submitted to iron-replete and iron-limited conditions. This work emphasizes that the stable expression of any potential reference candidate gene must be validated in each physiological condition or experimental treatment under study.

## Introduction

Reverse Transcription Quantitative real-time Polymerase Chain Reaction (RT-qPCR) has become the method of choice for gene expression quantification due to its simplicity, reproducibility, high sensitivity and specificity^1,2^. Relative quantification is the most common method for gene expression analysis, as it analyses the changes in expression of a target gene relative to the expression of a reference gene^3^.

Although accurate for gene expression analysis, RT-qPCR assays are subjected to several variables that can affect the reliability of the quantification, which include the initial amount of the sample, integrity and quantity of the extracted RNA, primer design and efficiency of cDNA synthesis^1,4^. The most accepted approach to minimize such variations is to perform a relative normalization, in which the expression level of a target gene is normalized relative to the expression level of an endogenous stably expressed gene, also called an internal control or reference gene^5–7^. The data normalization aim to correct for variations between cells and tissue types, culture conditions, and experimental treatment, thus allowing gene expression measurements to be compared across the samples^5,6^. For this reason, choosing an appropriate reference gene is crucial for relative gene expression analysis, since the accuracy of the expression data normalized relies on the stable expression of the reference gene.

A suitable endogenous control should have stable expression within the samples to be compared, regardless of physiological or experimental conditions^4^. It should also be expressed at roughly the same level as the mRNA level of the gene under study. Housekeeping genes are usually chosen for normalization of RT-qPCR data. Since these genes are involved in cellular basal metabolism, it is assumed that they are constitutively expressed. However, even housekeeping genes are subjected to expression variations, which imply that the stable expression of any potential reference candidate genes must be validated prior to its utilization on RT-qPCR normalization^8^. According to Vandesompele *et al*.^7^, it is recommended to use at least two reference genes to ensure more accurate and reliable normalization of gene expression analysis. Likewise, it is recommended that any endogenous control be validated in every physiological condition or experimental treatment under study, to ensure its stability in each particular situation^9^.

There is no consensus for an ideal and universal endogenous control for the opportunistic human pathogen *Klebsiella pneumoniae*. This bacterium is an important nosocomial pathogen responsible for wide range infections, including pneumonia, bacteremia, urinary and respiratory tract infections^10^. Nowadays, *K. pneumoniae* has become a threat worldwide^11^, mostly due to the emergence of nosocomial infections caused by multidrug-resistant *K. pneumoniae* isolates and to the spread of invasive infections, such as meningitis, endophthalmitis and liver abscess^12^. The virulence determinants of *K. pneumoniae* include the production of capsular polysaccharides, lipopolysaccharides, fimbrae and iron acquisition systems^12,13^.

In fact, iron is an essential element for most living organisms. In bacteria, iron is needed for growth and cellular metabolism, and it is also considered an important cofactor for regulation of many genes involved in bacterial virulence^14–16^. However, bacteria face iron scarcity when in contact to mammalian host, where the majority of the iron is intracellular and tightly bound to host iron-binding proteins such as transferrin, lactoferrin and hemoglobin^17^.

The predominant strategy employed by pathogens to acquire iron is through the production of siderophores, small iron-chelating molecules that exhibit high affinity for iron^13^. Several siderophores are expressed in *K. pneumoniae* strains, including enterobactin, yersiniabactin, salmochelin, and aerobactin^12^. The catecholate siderophore enterobactin has the highest affinity for iron and is considered the most prevalent siderophore-mediated iron acquisition system in *K. pneumoniae*^12,18,19^. Moreover, enterobactin has an affinity for ferric iron (Fe^3+^) greater than that exhibited by lactoferrin and transferrin and, therefore, can efficiently scavenge iron from the host. Once bound to Fe^3+^, the enterobactin-Fe^3+^ complex is captured and imported into the bacterium through its cognate outer membrane (OM) receptors. Gram-negative bacteria such as *K. pneumoniae* encode the OM receptors FepA, CirA and Fiu for catecholate-type siderophores transport^20^. FepA receptor display high ligand affinity to ferric enterobactin^21^, while CirA and Fiu exhibit specificity to ferric catecholates and their breakdown products containing ferric iron^22^. Since the transport of the iron-siderophore complex across the outer membrane is an active process, the siderophore receptors depend on the energy transduction system provided by the inner membrane complex TonB-ExbB-ExbD proteins^23^.

Although essential as a nutrient, excess of iron is toxic because of its ability to catalyze Fenton reactions that leads to generation of active species of oxygen^13^. Thus, bacteria have evolved an efficient mechanism for iron acquisition and maintenance of intracellular levels of this element^14,24^. In *K. pneumoniae*, like many other Gram-negative bacteria, iron homeostasis is regulated by the ferric uptake regulator (Fur)^25,26^. Fur protein binds ferrous iron (Fe^2+^) and other divalent metal cations, and the Fur-Fe^2+^ complex modulates gene expression by binding on consensus DNA sequences, known as Fur boxes, located in the promoter region of the target genes^25^. Fur also regulates the expression of virulence genes involved in motility, quorum sensing, stress resistance, toxin production, and biofilm formation^27,28^.

Despite the role of iron in *K. pneumoniae* pathogenicity, studies on the expression of iron-regulated virulence genes in this bacterium can be compromised since systematic investigations to establish reliable reference genes for *K. pneumoniae* have not yet been described. In this respect, we describe in the present study the selection and evaluation of eleven reference candidate genes for RT-qPCR gene expression analysis on *K. pneumoniae*. The expression of the candidate genes was assessed in *K. pneumoniae* under different culture media, non-ideal temperatures and various growth stages and the expression stability of the candidate genes was statistically evaluated with Bestkeeper, NormFinder, geNorm, Delta C_T_ and RefFinder programs. Finally, the validated reference genes were used to normalize the expression of iron-regulated genes in *K. pneumoniae* submitted to iron-replete and iron-limited conditions.

## Results

### Selection of candidate reference genes, PCR amplification efficiencies and expression profile

In this study, eleven genes (*aat*, *ffh*, *glnA*, *gyrA*, *proC*, *recA*, *rho*, *rpoC*, *rpoD*, *rrsH* and *trpS*) were selected and evaluated as potential candidate reference genes for RT-qPCR analyses in *K. pneumoniae*. Functional category, locus number, product name and function of each candidate gene are displayed on Supplementary Table S1.

The selected genes are highly conserved among distinct strains of *K. pneumoniae* from phylogenetic groups KpI, KpII and KpIII. As shown on Supplemental Tables S2 to S12, the nucleotide sequence identity between strains of the same phylogroup ranged from 98% to 100%, whereas sequence alignment between strains of different phylogroups showed sequence identity of 99% (*rrsH* gene, among all strains) to 91% (*aat* gene, KpI versus KpIII strains). The sequence alignments presented a query coverage ranging from 99% to 100% and the alignment of all sequences yielded e-values of 0.0 (data not show).

Table 1 shows the primer sequences specific for each selected candidate gene and the corresponding size of the amplicon for *K. pneumoniae* strain ATCC 10031. The amplification specificity was confirmed by the presence of a single PCR product of expected size on agarose gel electrophoresis (Supplementary Fig. S1). Dissociation-curve analyses revealed only single peaks indicating the absence of primer-dimers formation and nonspecific PCR products (Supplementary Fig. S2). Table 1 also displays that the PCR efficiencies ranged from 83.4% for *proC* to 98.2% for *glnA*, which is within the acceptable range for a reliable real-time PCR quantification. Furthermore, the standard curves revealed acceptable correlation coefficient (R^2^), thus confirming the reliability of the primer pairs in the RT-qPCR analysis.

**Table 1:** Selected candidate reference genes, their corresponding product name, primer sequences (annealing temperature of 60 °C), amplicon size in base pairs (bp), their respective PCR amplification efficiencies and the mean C_T_ values(±standard deviation) assessed in *K. pneumoniae* cells submitted to various experimental conditions and at different phases of growth.

Genes	Product Name	Forward and Reverse primer sequences (5′ > 3′)	Amplicon size	R^2^	E (%)	Mean C_T_ ± SD
*proC*	Pyrroline-5-carboxylate reductase	GATTGCCGATATCGTCTTCG GAGACCACCAGCGACTCTTT	99 bp	0.989	83.4	24.91 ± 0.62
*glnA*	Glutamine synthetase	GAAGGCGGTAACAAAGGTCA TACACATGGTGGAACGGATG	97 bp	0.988	98.2	23.79 ± 1.19
*gyrA*	DNA gyrase subunit A	GTGACCCGTCGTACGATTTT GATAATCGGGTCGATGTTGG	99 bp	0.987	96.6	24.45 ± 1.10
*recA*	Recombinase A	TTAAACAGGCCGAATTCCAG CCGCTTTCTCAATCAGCTTC	99 bp	0.989	92.1	22.40 ± 0.61
*rpoD*	RNA polymerase sigma factor RpoD	TCCGGTGCATATGATTGAGA ATACGCTCAGCCAGCTCTTC	105 bp	0.989	87.5	25.05 ± 0.80
*rho*	Transcription termination factor Rho	AACTACGACAAGCCGGAAAA ACCGTTACCACGCTCCATAC	99 bp	0.998	92.4	23.92 ± 0.90
*rpoC*	DNA-directed RNA polymerase subunit beta’	TATTCTGGTTCCACGCAACA GGATACAACGGAACGCACTT	97 bp	0.969	91.2	23.28 ± 0.88
*rrsH*	16S ribosomal RNA	GACGATCCCTAGCTGGTCTG GTGCAATATTCCCCACTGCT	95 bp	0.957	94.2	10.60 ± 0.55
*aat*	Leucyl/phenylalanyl-tRNA-protein transferase	CTGGATAACCAGCAGTATCGTTC GTACATTCCACCTACCAGCGTATT	106 bp	0.994	84.1	38.83 ± 1.55
*ffh*	Signal recognition particle protein	GCTAAGCCGGAAATCATCAA ATGTCGTCGAACTGCTTGAG	104 bp	0.986	97.2	23.93 ± 1.39
*trpS*	Tryptophanyl-tRNA synthetase II	GCCACTGTAAGGCGCTACTC GCCGATAACGTCAGCGTATT	100 bp	0.992	87.1	29.14 ± 1.50

R^2^: Correlation coefficient; E: PCR efficiency (%); S.D.: standard deviation.

The differences in transcript levels between the candidate genes are given as C_T_ values (mean ± standard deviation) on Table 1. Although the average C_T_ values ranged from 10 to 38, the majority of the candidate genes presented average C_T_ values between 22 and 24. *rrsH* (16S ribosomal RNA) was the most abundantly expressed gene in all of the samples (10.60 ± 0.55, mean CT ± standard deviation), whereas *aat* was the least abundantly expressed gene (38.83 ± 1.55). Overall, the selected genes presented minimum cycle variation across all samples, as indicated by the low standard deviation of the mean C_T_. Most genes showed cycle variation below 1 cycle and none above 2 cycles. *rrsH* and *recA* presented the least variation on cycle number (standard deviation of 0.55 and 0.61, respectively), and *ffh* and *aat* showed the largest variation on the cycle number (standard deviation of 1.39 and 1.55, respectively).

### Expression stability of candidate reference genes

The expression stability of the eleven candidate genes were assessed under various experimental conditions and evaluated using the statistical algorithms BestKeeper, geNorm, NormFinder, Delta C_T_ and RefFinder analyses.

Of the eleven candidate reference genes initially selected, two genes, *aat* and *trpS*, were excluded after preliminary analyses by BestKeeper and geNorm. According to BestKeeper, genes with standard deviation (SD values) greater than 1 are considered inappropriate as reference gene. BestKeeper analyses revealed that most of the selected candidate genes are suitable to be considered a reference gene since they had SD values lower than 1 (Supplementary Table S13). However, *aat* and *trpS* had the highest variable expression across all cultural conditions, with SD values of 1.56 and 1.50 respectively. Besides a great SD value, *trpS* also presented a coefficient of variation of the C_Ts_ (CV value) of 5.14% (Table S13) and, therefore, was considered the gene with the most expression variation. Furthermore, geNorm analysis indicated that *aat* and *trpS* presented expression stability value *M* of 1.19 and 1.10, respectively, which are above the threshold of ≤1.0 that indicates stability of gene expression. For these reasons, *aat* and *trpS* were considered unsuitable for a reference gene and were not included on further analysis.

The remaining nine genes were submitted to the Pearson correlation coefficient (symbolized by *r*) calculation by BestKeeper and to the subsequent statistical analyses with geNorm, NormFinder and RefFinder programs. Table 2 summarizes the statistical calculations generated by BestKeeper, geNorm, NormFinder, Delta C_T_ and RefFinder programs and shows the ranking order of the genes with the most stable expression across all different conditions tested.

**Table 2:** Expression stability ranking of the candidate reference genes according to BestKeeper, NormFinder and geNorm original softwares and the Delta C_T_ and RefFinder analysis.

Ranking	BestKeeper		NormFinder		GeNorm		Delta C_T_ analysis		RefFinder analysis	
	Gene	*r (p-value)*	Gene	Stability Value *(standard error)*	Gene	*M* value	Gene	Average of STDEV	Gene	Geometric mean of ranking values
1	*recA*	0,849 (*0.001*)	*recA*	0,296 (*0.082*)	*rpoC*	0,44	*recA*	0,92	*recA*	1.78
2	*rho*	0,816 (*0.001*)	*rho*	0,328 (*0.085*)	*proC*	0,44	*rho*	0,92	*rpoD*	2.99
3	*glnA*	0,814 (*0.001*)	*proC*	0,423 (*0.096*)	*rpoD*	0,49	*rpoD*	1	*rho*	3
4	*gyrA*	0,739 (*0.001*)	*rrsH*	0,515 (*0.109*)	*rrsH*	0,6	*gyrA*	1,01	*rpoC*	3.13
5	*ffh*	0,712 (*0.002*)	*gyrA*	0,535 (*0.112*)	*recA*	0,67	*proC*	1,02	*proC*	3.31
6	*rpoD*	0,687 (*0.003*)	*rpoC*	0,614 (*0.124*)	*rho*	0,78	*rpoC*	1,06	*rrsH*	4.28
7	*proC*	0,66 (*0.005*)	*rpoD*	0,674 (*0.134*)	*gyrA*	0,86	*rrsH*	1,08	*gyrA*	6.19
8	*rpoC*	0,541 (*0.03*)	*ffh*	0,76 (*0.148*)	*glnA*	0,92	*glnA*	1,12	*glnA*	7.74
9	*rrsH*	0,42 (*0.106*)	*glnA*	0,776 (*0.15*)	*ffh*	0,99	*ffh*	1,31	*ffh*	9

As stated by BestKeeper program, the higher the coefficient of correlation (*r*), the greater the stability of the gene. As shown in Table 2, BestKeeper indicated *recA* (*r* = 0.849), *rho* (*r* = 0.816) and *glnA* (*r* = 0.814) with the largest *r* value and *p*-value of 0.001. Thus, these genes showed the most stable expression and hence they are the most suitable genes for endogenous control. Although *rrsH* gene showed stable expression in all tested conditions – as indicated by the least variation on C_T_ number (mean 10.60 ± 0.55 standard deviation, Table 1) – this gene presented a high CV value (4.10%) and the lowest correlation coefficient (*r* = 0.42, *p*-value of 0.106), which resulted in the ninth position to *rrsH* gene in the BestKeeper ranking (Table 2).

GeNorm analysis of the candidate reference genes rendered an average expression stability *M* values below the threshold of 1.0, indicating that all tested genes have stable expression across samples (Table 2). The analysis indicated *proC* (0.44), *rpoC* (0.44) and *rpoD* (0.49) with *M* values bellow 0.5 and hence they were considered the top most stable genes (Table 2). *rrsH* (0.60), *recA* (0.67) and *rho* (0.78) were estimated to have intermediary *M* values, while *gyrA* (0.86), *glnA* (0.92) and *ffh* (0.99) exhibited the highest *M* values among all genes analyzed, but still below 1.0. Accordingly, these genes also had stable expression and are suitable as normalization factor in RT-qPCR analysis.

**Figure 1:**
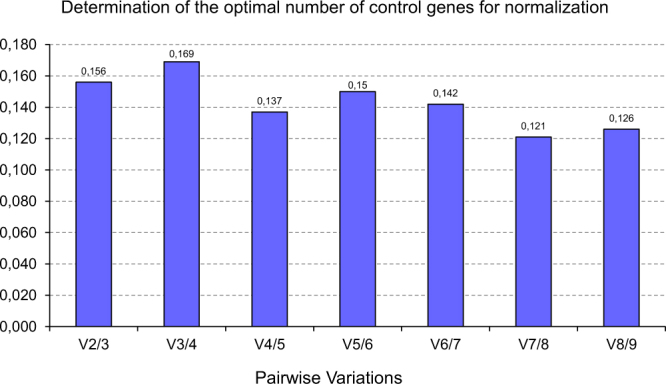
Optimal number of reference genes for normalization of RT-qPCR data indicated by geNorm analysis. GeNorm calculates the pairwise variation (V_n_/V_n+1_, V value) between the normalization factors NF_n_/NF_n+1_ to determine the minimum number of reference genes required for accurate normalization. Vandesompele and colleagues^7^ suggest a cut-off threshold of 0.15, below which the inclusion of another reference gene is not required. The V_4/5_ value of 0.137 indicates that the inclusion of the fifth most stable reference gene (*recA*) would have no significant contribution to the accuracy of normalization.

GeNorm also calculates pairwise variation (V value) to determine the optimal number of reference genes required for accurate normalization of RT-qPCR data, based on the recommended V value threshold of 0.15. As shown in Fig. 1, the V value drops below the recommended 0.15 cut-off only when the fifth most stable reference gene (*recA*) is included (V_4/5_ of 0.137). This result indicates that the top four reference genes (*proC*, *rpoC*, *rpoD* and *rrsH*) would be adequate to ensure accurate normalization of RT-qPCR data, and that the inclusion of *recA* would have no significant contribution to the accuracy of normalization. However, as emphasized by the geNorm authors^7^ the proposed value should not be taken as a too strict threshold but rather is intended to be guidance for determination of the optimal number of reference genes.

According to the NormFinder program, genes with the lowest stability values present the slightest change in expression. As shown in Table 2, NormFinder indicated *recA*, *rho* and *proC* as the top ranked genes with stability values of 0.296, 0.328 and 0.423, respectively. *rrsH* (0.515), *gyrA* (0.535), *rpoC* (0.614) and *rpoD* (0.674) had intermediary stability values, while *ffh* (0.760) and *glnA* (0.776) were ranked the least stable genes with the highest stability values. NormFinder also indicated the best combination of two genes, which were *proC* and *rho* with a stability value of 0.185.

The online available RefFinder tool was also used to compare and rank the expression stability of the candidate reference genes. As displayed on Table 2, the recommended comprehensive ranking by RefFinder indicates *recA*, *rpoD* and *rho* as the most suitable genes for endogenous control, with geometric mean of ranking values of 1.78, 2.99 and 3.0, respectively. Moreover, Delta C_T_ analysis performed by RefFinder shows *recA*, *rho* and *proC* as the top ranked genes, with average of standard deviation (STDEV) of 0.92, 0.92 and 1.0, respectively.

Supplementary Table S14 compares the BestKeeper, geNorm and NormFinder results calculated by RefFinder with those calculated by their corresponding original softwares. The BestKeeper ranking and stability values were exactly the same, no matter if the analysis were executed by the original BestKeeper software or by RefFinder tool. Concerning geNorm analysis, the only difference observed when the analysis were perfomed by the original software or by RefFinder tool was a shift between *rpoD* and *proC* on the second and third position on the rank. On the other hand, comparison of the NormFinder results perfomed by the original software and the RefFinder tool reveals similar results only among the top three ranking genes (Table S14).

### Identification of Fur-regulated genes

Bioinformatics analyses were carried out to identify putative Fur-binding sites on the promoter region of genes related to iron-acquisition systems in *K. pneumoniae*. Table 3 shows the putative Fur boxes identified on the upstream region of the genes *cirA*, *iroN* and *fiu*, which encode the catecholate-type siderophore receptors CirA, FepA, and Fiu. To determine whether these new putative Fur boxes are functional, two assays were executed: Fur Titration Assay (FURTA) and DNA Electrophoretic Mobility Shift Assay (EMSA).

**Table 3:** Putative Fur boxes identified on the promotor region of genes related to iron-acquisition systems.

Genes	Product Name	Putative FUR BOX		
		Location^a^	Sequence^b^	Score^c^
*cirA*	Colicin I receptor and catecholate siderophore receptor	242 bp	GATAATGATTACGATTATC	21.80
*iroN*	Outer membrane receptor FepA	48 bp	TGTAATGATAATTGTTATC	17.98
*fiu*	Catecholate siderophore receptor Fiu	107 bp	GCAAATGATAACTATTCTT	16.17

^a^Location in base pairs (bp) upstream of the start codon. ^b^Nucleotides identical to the proposed Fur-binding consensus sequence^25^ are underlined. ^c^Score expressed in bits.

On FURTA, *E. coli* strain H1717, which harbors the *lacZ* reporter gene under control of the Fur-regulated *fhuF* gene promoter, was transformed with the putative Fur boxes cloned in a high copy number vector. If the putative Fur boxes were functional, *E. coli* Fur repressor is titrated away from the promoter_*fhuF*_::*lacZ* gene fusion, thus releasing the expression of *lacZ*. The product of this gene, beta galactosidase, will render red *E. coli* H1717 colonies on MacConkey lactose agar plates under iron-rich condition. If the putative Fur boxes were not functional, *E. coli* Fur repressor remains bound on promoter_*fhuF*_::*lacZ* gene fusion and, in the absence of beta galactosidase, colorless *E. coli* H1717 colonies will appear on the MacConkey agar plates.

**Figure 2:**
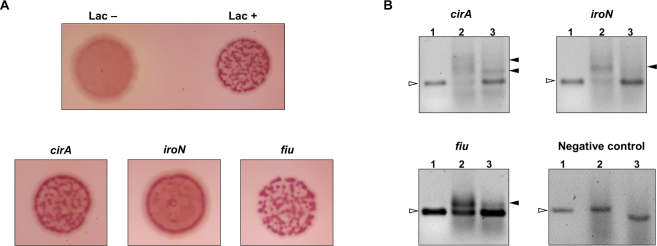
Validation of the putative Fur boxes identified on the upstream region of the genes *cirA*, *iroN* and *fiu* by FURTA (**A**) and EMSA (**B**). In (**A**), Lac^−^ indicates FURTA-negative phenotype, whereas Lac^+^ indicates FURTA-positive phenotype. All putative Fur boxes resulted on red *E. coli* H1717 colonies on MacConkey plates, which were interpreted as FURTA positive results. In (**B**), EMSA of the DNA fragments containing the putative Fur box of *cirA*, *iroN*, *fiu* and the negative control (DNA fragment without Fur box). Lanes 1, 2 and 3 contained 50 ηg of the respective DNA probes. The DNA probes were incubated with 500 ηM of His-Fur protein either in the presence of divalent cation (Lanes 2) or under divalent cation-free conditions by adding 2 mM EDTA (Lanes 3). Open arrowheads indicate the free DNA probes, while closed arrowheads indicate the mobility shift corresponding to the Fur/DNA complexes. Full-length gels are presented in Supplementary Fig. S3.

Figure 2A shows that all putative Fur boxes identified on this study rendered red *E. coli* H1717 colonies on MacConkey plates, which confirms that *E. coli* Fur repressor was able to bind the cloned putative Fur boxes in an iron-dependent manner.

To confirm the results observed on FURTA, EMSA was performed to confirm whether *K. pneumoniae* Fur protein directly binds on the putative Fur boxes. As shown on Fig. 2B, the *K. pneumoniae* purified His-Fur protein was able to interact with the DNA fragments containing the putative Fur boxes in the presence of divalent Manganese ions (Mn^2+^), which resulted on a mobility shift of the DNA fragments. Addition of EDTA to the binding reaction mixture abolished the mobility shift of the fragments, indicating that divalent cations are required for Fur interaction with the promoter regions of *cirA*, *iroN* and *fiu* genes. The negative control, which consisted of a 254 base pairs DNA fragment without Fur box sequence, was unable to complex with the *K. pneumoniae* purified His-Fur protein, and a mobility shift was not observed (Fig. 2B).

### Expression profile of Fur-regulated genes normalized with the candidate reference genes

To evaluate the reliability of the candidate reference genes, the top two most stable reference genes, *recA* and *rho*, were selected to normalize the relative expression levels of the Fur-regulated genes *cirA*, *iroN* and *fiu* in *K. pneumoniae* submitted to iron-replete and iron-limiting conditions. RT-qPCR data were also normalized with *ffh* gene, which was considered the least stable gene, and with *rrsH* gene, which is commonly used as endogenous control.

**Figure 3:**
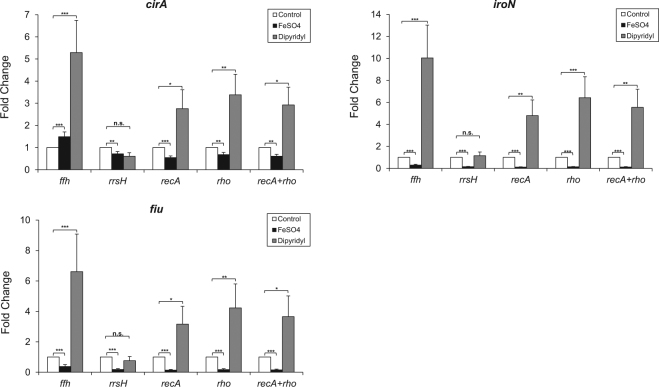
Relative expression of the Fur-regulated genes *cirA*, *iroN* and *fiu* in *K. pneumoniae* cells submitted to iron-replete (FeSO4) and iron-limited (Dipyridyl) conditions. The expression data were normalized using *ffh*, *rrsH*, *recA* or *rho* as reference genes individually and with the geometric mean of *recA* + *rho*. Adjusted *p*-value are indicated as following: *p < 0.05, **p ≤ 0.01 and ***p ≤ 0.001. N.S.: non-significant *p*-value.

As shown in Fig. 3, normalization of RT-qPCR data using the two most stable reference genes, *recA* and *rho*, revealed upregulation of *cirA*, *iroN* and *fiu* in iron-limiting condition and downregulation in iron-replete condition, when compared to the control condition. Similar results were obtained when the data were normalized with *recA* and *rho* individually and with the *recA* + *rho* combined as a normalization factor.

On the other hand, normalization with *ffh* and *rrsH* yielded inconsistent results (Fig. 3). When the least stable *ffh* gene was used, *cirA* appears upregulated at both iron-replete and iron-limiting conditions. Moreover, normalization with *ffh* resulted in almost twofold increase in the upregulation of *cirA*, *iroN* and *fiu* in the presence of the iron chelator, compared to the expression profile of these genes normalized with *recA* and *rho*. When *rrsH* was used as normalizer, no significant difference in the expression pattern of *cirA*, *iroN* and *fiu* was observed at iron-limiting conditions (Fig. 3).

## Discussion

*Klebsiella pneumoniae* is an important nosocomial pathogen that has recently become a global threat due to the emergence and spread of hypervirulent and antibiotic-resistant strains presenting elevated morbidity and mortality^12^. An important aspect of the studies of *K. pneumoniae* pathogenicity is the expression analysis of virulence-related genes by Reverse Transcription Quantitative real-time Polymerase Chain Reaction (RT-qPCR). One of the most critical steps on RT-qPCR analysis is the choice of the reference gene with which the data will be normalized. The ideal reference gene should have stable expression regardless of the physiological state and with minor variations during experimental conditions. However, there is no ideal reference gene that meets the mentioned criteria. This implies that any potential reference candidate genes must be validated in every physiological condition or experimental treatment under study prior to its utilization on RT-qPCR normalization^9^.

Unfortunately, there is no validated reference gene for *K. pneumoniae* described in the literature. Several genes have been used as normalizer in gene expression analysis on *K. pneumoniae*, although they did not have their expression stability properly validated. For instance, RT-qPCR analysis in *K. pneumoniae* has been normalized by using either standard curve^29^ or a diversity of genes, such as *uncB*^30^, *rfaH*^31^, *rpoB*^32,33^, *rpoD*^34,35^ and ribosomal RNA coding genes. In fact, the most extensively used reference gene in RT-qPCR analysis in *K. pneumoniae* are the genes encoding the 16S^36–39^ and 23S^40–42^ ribosomal RNAs. However, there are restrictions on the use of a ribosomal RNA for the normalization of messenger RNAs. Firstly, ribosomal RNA genes are not recommended for RT-qPCR analysis due to the high abundance of transcripts from this gene, which hinder the quantification of rare and less abundant mRNA transcripts^43^. This high abundance also requires the dilution of the cDNA samples prior to RT-qPCR reactions, thus increasing the risks of dilution errors^7^. Secondly, ribosomal and messenger RNAs present distinct lifetimes within the cell, which difficult comparison among them. While messenger RNAs show rapid turn-over according to the physiological conditions of a bacterium, ribosomal RNA is only degraded under certain stress conditions^44^. Thirdly, some studies have suggested that the expression of ribosomal RNA genes may be under some regulatory control^45–47^, which goes against the constitutive expression characteristic that is essential for any endogenous control.

The lack of suitable reference genes for gene expression analysis in *K. pneumoniae* prompted us to perform a systematic approach to identify and validate reliable reference genes to be used on RT-qPCR analysis in this bacterium.

In this study, we selected and evaluated eleven candidate reference genes (*aat*, *ffh*, *glnA*, *gyrA*, *proC*, *recA*, *rho*, *rpoC*, *rpoD*, *rrsH* and *trpS*) commonly used in different bacterial species as potential reference genes for *K. pneumoniae*. Despite the heterogeneous population of pathogenic *K. pneumoniae*^11^, the selected candidate genes are highly conserved among strains from the distinct *K. pneunoniae* phylogroups KpI, KpII and KpIII. The expression profile of the candidate genes was assessed in *K. pneumoniae* cells submitted to various experimental conditions and at different phases of growth. Then, the expression stability of the candidate genes was calculated using the statistical algorithms BestKeeper, NormFinder, geNorm, Delta C_T_ and RefFinder analyses.

The *rrsH* gene encoding the 16S subunit of ribosomal RNA presented stable expression in all conditions tested. However, this gene showed high coefficient of variation and the lowest correlation coefficient among the genes analyzed by BestKeeper program. Besides, the high abundance of transcripts from this gene, indicated by the lowest C_T_ number among all genes analyzed, hinders the use of this gene as endogenous control. These results suggest that *rrsH* gene should be discarded as reference gene for RT-qPCR analysis in *K. pneumoniae*. Similar conclusions were reached by other authors for other bacteria. For instance, the transcript levels of the *rrs* gene (encoding 16S rRNA) in the lactic acid bacterium *Oenococcus oeni* were 1000-fold higher than the transcript levels of mRNAs^43^. The authors concluded that this gene should not be chosen as internal control for this bacterium. Takle *et al*.^48^ showed that the16*S* gene was not stably expressed in the tested culture conditions, thus suggesting that *16*S is inappropriate as a reference gene in gene expression studies in the plant pathogen *Pectobacterium atrosepticum*. According to Nieto *et al*.^49^, the *rrs* gene showed stable expression under the conditions tested, but it is not recommended as a reference gene in the extremophile *Acidithiobacillus ferrooxidans* because its more abundant expression prejudices the measurements of low abundance transcripts. Badejo *et al*.^50^ discarded the *rrs* gene as an internal control for *Mycobacterium gilvum* since the expression of this gene was highly unstable in the experimental conditions analyzed.

Although several studies have indicated that genes encoding ribosomal RNAs are not reliable as internal control, many studies show that these genes can be considered a good reference gene in some bacteria, such as *Moraxella catarrhalis*^51^, *Clostridium difficile*^52^ and *Gluconacetobacter diazotrophicus*^53^. Therefore, before a ribosomal RNA gene be discarded as endogenous control, it is recommended that the expression stability of these genes be tested under various physiological and experimental conditions, and that its elevated expression level be considered and adjusted to avoid possible bias.

The top two most stably expressed genes determined by BestKeeper, NormFinder and Delta C_T_ analyses were *recA* and *rho*. RefFinder also indicated *recA* as the top stable gene, but ranked *rho* as the third most stable. Besides *recA* and *rho*, *proC* was ranked among the top three most stable genes by NormFinder and geNorm, and *rpoD* was ranked among the top three reference genes by geNorm, Delta C_T_ and RefFinder programs.

GeNorm’s pairwise variation analysis indicated the use of the top four reference genes (*proC*, *rpoC*, *rpoD* and *rrsH*) as the minimum number of genes required to ensure accurate normalization of RT-qPCR data. However, the other statistical programs revealed that *rpoC* is not among the top most stable genes and that *rrsH* was considered unsuitable as reference gene in *K. pneumoniae*. Therefore, we suggest that they should be excluded and, instead, replaced by *recA* and *rho* in the normalization factor.

The BestKeeper, NormFinder and geNorm analyses carried out by RefFinder yielded results with slight differences when compared to the results obtained by the three softwares individually. Although this result seems to validate the RefFinder tool, caution should be taken when employing and interpreting RefFinder outputs. This web-based platform utilizes raw C_T_ values and does not take into account the efficiency of primers, which may bias the final ranking of the candidate reference genes.

Taken all statistical analysis into consideration we suggest *recA*, *rho*, *rpoD* and *proC* as the most suitable reference genes for normalization of RT-qPCR data in *K. pneumoniae*. Similar to our findings, these reference genes were also indicated as the most suitable internal control genes for RT-qPCR data normalization in other bacteria, such as in *Actinobacillus pleuropneumoniae*^54^, *Erwinia amylovora*^55^, *Gluconacetobacter diazotrophicus*^53^, *Pectobacterium atrosepticum*^48^, *Pseudomonas aeruginosa*^56^ and *Staphylococcus aureus*^57^. Supplemental Tables S15 to S26 display the primer sequences of the four proposed reference genes for distinct strains of *K. pneumoniae* from phylogroups KpI, KpII and KpIII.

To test the strength and reliability of the proposed reference genes, we applied *recA* and *rho* as reference genes to normalize the relative expression of genes related to iron uptake systems in *K. pneumoniae* cells submitted to iron-replete and iron-limited conditions. We decided to test the proposed reference genes under such conditions because iron availability is crucial for both survival and virulence of pathogenic bacteria.

To acquire iron, *K. pneumoniae* synthesizes and secretes high-affinity iron-chelating molecules known as siderophores. A number of studies have shown that the production of multiple iron uptake systems enhances the pathogenicity of *K. pneumoniae* clinical isolates and that hypervirulent strains secretes more active siderophore molecules than non-hypervirulent strains^19,58–60^. Enterobactin, a catecholate-type siderophore, is considered the primary iron acquisition system utilized by *K. pneumoniae* since is almost ubiquitous among classical and hypervirulent strains^12^. However, this siderophore is inhibited by the host molecule lipocalin-2 (LCN2). To evade LCN2, some strains of *K. pneumoniae* produce salmochelin, a glycosylated form of enterobactin that it is not neutralized by LCN2^12^. In Gram-negative bacteria, including *K. pneumoniae*, siderophore-bound iron is captured by specific outer membrane receptors and it is actively transported into the cytoplasm to be used by the cell^20^. Salmochelin is captured by the outer membrane receptor FepA specifically encoded by *iroN* gene, while CirA and Fiu receptors, encoded by *cirA* and *fiu*, uptake ferric catecholates and their degradation products containing ferric iron^20,22^.

In *K. pneumoniae* the iron homeostasis is controlled by the ferric uptake regulator (Fur protein), which acts as a transcriptional repressor of iron-regulated genes. In its classical mechanism of action, Fur complexes with iron cofactor under iron-rich conditions and this complex binds to consensus DNA sequences, named Fur boxes, located in the promoter region of the target genes. Binding of Fur at the promoters prevents the binding of RNA polymerase and the transcription of the target gene^27^.

Here we described the identification and validation of Fur boxes on the promoter region of *cirA*, *iroN* and *fiu*, thus suggesting that the expression of these genes in *K. pneumoniae* is regulated by Fur repressor in an iron-dependent manner. To confirm these results, RT-qPCR analysis were employed to access the expression of *cirA*, *iroN* and *fiu* in *K. pneumoniae* cells subjected to iron-rich and low-iron conditions. When normalized with *recA* and *rho* reference genes, *cirA*, *iroN* and *fiu* appear upregulated and downregulated respectively in iron-replete and iron-restricted conditions. On the other hand, inconsistent results were obtained when *ffh* and *rrsH* were used as normalizers. These results validate the use of *recA* and *rho*, either individually or as a combined normalization factor, as reference genes in *K. pneumoniae* and highlight how the use of unsuitable reference genes can compromise RT-qPCR data normalization.

The differentially expression of *cirA*, *iroN* and *fiu* under iron-rich and low-iron conditions is consistence to the fact that these genes are regulated by Fur repressor and that ferrous iron acts as a corepressor. This is the first report describing the Fur-mediated regulation of *cirA*, *iroN* and *fiu* genes in *K. pneumoniae*, although the iron-dependent regulation of these genes has already been described in other enterobacteria. For instance, in *E. coli* the expression of *cirA*^61,62^, *fepA*^61,63,64^ and *fiu*^61^ are higher under iron-depleted conditions than under iron-replete conditions. Upregulation of *cirA* under iron starvation has also been described in *Salmonella typhimurium*^65^.

It is widely accepted that using at least two reference genes is sufficient to ensure high quality data, and that three reference genes would be ideal. According to the MIQE Guidelines^1^, normalization against a single reference gene is only acceptable if the gene presents invariant expression under the experimental tested conditions. Here we showed that *recA* and *rho* were considered, by the majority of the statistical programs, the genes with the most stable expression across all different conditions tested. Moreover, the normalization of RT-qPCR data using either *recA* and *rho* individually or as a combined normalization factor resulted in similar and reliable results. Despite this, to guarantee an accurate normalization of RT-qPCR data we suggest the use of at least two endogenous control among the four proposed reference genes.

In summary, the reliability of RT-qPCR analyses depend on accurate data normalization, which can only be achieved by using validated reference genes. Ideally, the stable expression of any potential reference candidate gene must be tested in every experimental treatment and condition under study. In this study, we recommend *recA*, *rho*, *rpoD* and *proC* as reference genes for gene expression normalization in *K. pneumoniae*. It is worth mentioning that the heterogeneous nature of the *K. pneumoniae* population should be taken into account when using the proposed reference genes. It is advisable to assess the expression stability of the proposed reference genes in a given strain under study, prior to their use on RT-qPCR data normalization. To the best of our knowledge, this is the first systematic study aimed to identify reference genes for RT-qPCR analyses in this bacterium.

## Methods

### Selection of candidate reference genes and design of primer pairs

Potential candidates for endogenous reference genes for *Klebsiella pneumoniae* were selected based on internal controls commonly used in other bacteria, as described on the literature. The selected candidate genes included: *aat* (leucyl/phenylalanyl-tRNA-protein transferase)^49^, *ffh* (signal recognition particle protein)^48^, *glnA* (glutamine synthetase)^48^, *gyrA* (DNA gyrase subunit A)^43,48,52,57^, *proC* (pyrroline-5-carboxylate reductase)^43,48,52,57^, *recA* (recombinase A)^48,49,53^, *rho* (transcription termination factor Rho)^22,48,52,53,57^, *rpoC* (DNA-directed RNA polymerase subunit beta’)^49,53^, *rpoD* (RNA polymerase sigma factor RpoD)^43,53,57^, *rrsH* (16S ribosomal RNA)^43,48,52,57^ and *trpS* (tryptophanyl-tRNA synthetase II)^49^. To minimize the chance of coregulation among the selected genes, the selection was performed so that the candidate genes belonged to different functional categories, such as cell metabolism (*proC* and *glnA*), protein synthesis (*rrsH*, *aat*, *ffh* and *trpS*), DNA replication (*gyrA* and *recA*) and transcription (*rpoC*, *rpoD* and *rho*). To check the homology of the selected candidate genes among different strains of *K. pneumoniae*, we perfomed BLAST multiple alignment of the nucleotide sequence of each gene on strains from the three phylogroups of *K. pneumoniae*: KpI (strains 1084, ATCC 10031, CG43, JM45, HS11286, KCTC 2242, Kp13, NTUH-K2044 and MGH 78578), KpII (strains ATCC 700603 and HKUOPA4) and KpIII (strain 342).

Primer pairs for each gene were designed based on the entire coding region of the candidate genes. Primers were designed using Primer3 *v. 0.4.0*^66^ according to the following parameters: primer length of about 20 bases, GC content of 45–60%, melting temperature (Tm value) of 60 °C, and amplicon size ranging from 95 to 105 base pair. Prior to the Real-time PCR assays, all primer pairs were tested by conventional PCR followed by agarose gel electrophoresis, in order to check for specificity of PCR amplification. The amplicons were visualized under UV light and recorded with a digital photodocumentation system (Gel Doc^TM^ XR, Biorad). The imagens were captured and analyzed with the Image Lab^TM^ Software version 5.0 (Biorad).

### Bacterial strain and growth conditions

*K. pneumoniae* strain ATCC 10031 was cultured in LB liquid medium on a rotary incubator shaker (150 rpm) at 37 °C under aerobic conditions. Bacterial growth was monitored by measuring the optical density of the cultures at a wavelength of 600 nm (O.D._600 nm_), using the GeneQuant Spectrophotometer (GE Healthcare).

The expression of the candidate reference genes was analyzed in *K. pneumoniae* cells submitted to various experimental conditions and at different phases of growth. Regarding the phases of growth, bacterial cells were harvested in the lag (O.D._600 nm_ = 0.2), exponential (O.D._600 nm_ = 0.6) and stationary (O.D._600 nm_ = 1.9) stages. For conditions of thermal stress, bacterial cells at the exponential phase were incubated for 30 minutes at 29 °C (cold shock) or 45 °C (heat shock) and then harvested. The expression of the candidate genes was also analyzed under different availability of iron in the culture medium. In this case, cells at the exponential phase were harvested 30 minutes after the addition of a source of ferrous iron (FeSO_4_, Sigma-Aldrich) or an iron chelator (2,2′-Dipyridyl, Sigma-Aldrich) in LB medium to a final concentration of 100 μM. In addition, bacterial cells at the exponential phase were harvested 30 minutes after the addition of MnSO_4_ in the culture medium (100 μM, final concentration). The expression of the genes were assessed in the presence of MnSO_4_ since this compound is commonly used as a source of divalent ions (Mn^2+^) for studies of Fur-mediated iron regulation, in replace of the highly oxidizable ferrous iron. All culture conditions were done in triplicates. Bacterial cells were harvested by centrifugation, the cell pellets were ressuspended in *RNAprotect*^*®*^ *Bacteria Reagent* for RNA stabilization as described by the manufacturer (Qiagen) and immediately submitted to total RNA extraction.

### Total RNA extraction and first strand cDNA synthesis

Total RNA was extracted using *RNeasy Mini Kit* (Qiagen), as recommended by the manufacturer’s protocol. An on-column DNase digestion with the RNase-free DNase Set (Qiagen) was included to remove any genomic DNA contamination in RNA samples. RNA integrity was analyzed by agarose gel electrophoresis and its purity and concentration were calculated by measuring the optical density of the samples at 260 and 280 ηm using a spectrophotometer.

For single strand cDNA synthesis, 1 μg of high quality purified RNA was reverse transcribed in a 20 μL volume reaction using random hexamers and *ThermoScript™ RT-PCR System*, according to the manufacturer’s instructions (Invitrogen). Prior to use on Real-time PCR assays, the synthesized cDNA samples were diluted with nuclease-free water to a final concentration of 100 ηg/μL (roughly a 1:20 dilution).

### Real-time PCR assays and verification of PCR amplification efficiency

The Real-time PCR assays were carried out in a 7300 Real-Time PCR System (Applied Biosystems) using the *Platinum*^*®*^ *SYBR*^*®*^ *Green qPCR SuperMix-UDG* (Thermo Fisher Scientific), following the manufacturer’s instructions. Reaction mixture consisted of 1 μL of diluted cDNA, 400 ηM of each primer, ROX reference dye at final concentration of 500 ηM and 1x SYBR Green qPCR SuperMix adjusted with nuclease free water to a final volume of 12,5 μL. Due to high abundance of 16S ribosomal RNA subunit, the expression of *rrsH* gene was measured using cDNA diluted 200 fold. The RT-qPCR reactions were done in triplicate for each cDNA sample.

The reactions were initially incubated at 50 °C for 2 minutes for Uracil-DNA glycosylase treatment, followed by denaturation for 2 minutes at 95 °C. After this pretreatment, reactions were subjected to the following thermal cycling conditions: 40 cycles of denaturation at 95 °C for 15 seconds and annealing/extension at 60 °C for 60 seconds. Finally, dissociation (melting) curve analyses were performed to check for nonspecific amplification and/or primer-dimers formation.

The Real-time PCR data were detected and analyzed by the software *7300 Real-Time PCR System Sequence Detection Software* v1.4.1 (Applied Biosystems) according to default parameters, which generated the Cycle Threshold (C_T_) values for each reaction. The average C_T_ number of each triplicate Real-time PCR reactions was used on the subsequent statistical analyses.

Standard curves were constructed for every candidate reference gene to determine the PCR amplification efficiency and the regression coefficient (R^2^) of each pair of primers. Ten-fold serial dilution of genomic DNA from *K. pneumoniae* was used in Real-time PCR reactions and the five-point standard curves were generated by plotting the average C_T_ numbers versus the logarithm of the amount of template DNA. PCR amplification efficiency (E) of each primer pair was calculated from the linear regression and the slope of the corresponding standard curves, according to the formula: E (%) = [10^(−1/slope)^ − 1] × 100. The efficiency of each reference gene was considered in all subsequent statistical analysis.

### Reference gene expression stability analyses

The expression stability of the selected reference genes was statistically evaluated by three commonly used Microsoft Excel-based softwares: BestKeeper^67^, geNorm^7^ and NormFinder^68^. BestKeeper analyses allow the input of raw C_T_ values, while geNorm and NormFinder require that the raw C_T_ values be converted to relative quantification data. To achieve this, the raw C_T_ values were transformed into relative quantities using the formula 2^(−ΔCT)^, in which ΔC_T_ corresponds to the highest C_T_ value minus all other C_T_ value for each reference gene measured across all samples.

BestKeeper relies on the coefficient of variation (CV [% C_T_]) and the standard deviation (SD) of the C_T_ values (SD [±C_T_]) to estimate the expression stability of the candidate genes. According to BestKeeper program, the genes can be ranked from the most stably expressed, exhibiting the lowest SD [±C_T_], to the least stably expressed, exhibiting the highest SD [±C_T_]. Genes with SD greater than 1 are considered inconsistent by BestKeeper. Furthermore, BestKeeper performs numerous pairwise correlation analyses between each gene analyzed and the geometric mean C_T_ value of all the candidate genes together. Within each such pairwise correlation the program calculates the Pearson correlation coefficient, symbolized by *r*, and the probability *p*-values^67^. According to BestKeeper, the higher the correlation coefficient (*r*), the greater the stability of gene expression.

GeNorm ranks the candidate reference genes based on the expression stability value *M*, which is defined as the average pairwise variation between a particular reference gene and all other reference genes tested. Genes with the lowest *M* values are considered to have the most stable expression under tested experimental conditions and genes with *M* values above the threshold of 1.5 are not acceptable as reference genes^7^. In an even more stringent analysis, several studies have established the threshold of *M* value ≤ 1.0 to identify the most suitable reference genes for RT-qPCR normalization^48,54,69,70^. Stepwise exclusion of the least stable gene (with the highest *M* value) allows ranking of the tested genes according to their expression stability in the tested samples.

GeNorm also performs pairwise variation analysis to determine the optimal number of reference genes required for accurate normalization. This is achieved by calculating the pairwise variation value (V_n_/V_n+1_ value) between sequential normalization factors containing increasing number reference genes (NF_n_/NF_n+1_). Vandesompele and colleagues^7^ recommend to add additional reference genes to the normalization factor until the V_n_/V_n+1_ (V) value reaches the cut-off threshold of 0.15. V value below 0.15 means that the inclusion of an additional reference gene is not necessary since will not improve the normalization accuracy.

NormFinder considers not only the overall expression variation of the candidate reference genes but also the intra- and inter-group expression variations to calculate the stability value for each candidate gene^68^. Therefore, NormFinder ranks the candidates with minimal estimated variation between sample subgroups of the sample set. According to the analysis, the top ranked genes are those with the smallest stability value and the best combination of two reference genes is also indicated.

The expression stability of the selected reference genes was also evaluated by RefFinder^71^, available on the website http://leonxie.esy.es/RefFinder/. RefFinder is a web-based tool that provides a recommended comprehensive ranking based on the geometric mean of ranking values. RefFinder also integrates BestKeeper, geNorm and NormFinder softwares, and the comparative Delta C_T_ method^72^ to compare the expression stability of the candidate reference genes. The raw C_T_ values are directly input into the program to calculate and rank the tested candidate reference genes.

### Identification of putative Fur-regulated genes

Since iron is a crucial cofactor for regulation of virulence genes expression in many pathogenic bacteria, we decided to test the strength and reliability of the proposed reference genes on relative expression normalization of iron-regulated genes in *K. pneumoniae* cells submitted to iron-replete and iron-limited conditions. To find iron-regulated genes, bioinformatics analysis were employed on the genomic sequence of *K. pneumoniae* strain ATCC 700721/MGH 78578 (GenBank accession number CP000647.1) to identify putative Fur-binding sites on the promoter region of genes related to iron-acquisition systems in this bacterium. These analyses were conducted according to a theoretical approach described elsewhere^73^ and adapted to *K. pneumoniae*. In brief, a set of experimentally confirmed Fur boxes from *K. pneumoniae*^40,42,74^ was used to create a position weight matrix (PWM) model. This matrix was employed to search the *K. pneumoniae* genomic region where genes related to iron-acquisition systems are located. A 19 bp sliding window was used in this search. Only windows with scores, in the Fur weight matrix, higher than 7 bits were retained in the analysis. Complementary oligonucleotides containing the sequences of the putative Fur boxes were annealed to form double-stranded DNA probes. These DNA probes were used in Fur titration assay (FURTA) and DNA electrophoretic mobility shift assay (EMSA), in order to validate the Fur interactions with the putative Fur boxes.

### Fur Titration Assay (FURTA)

FURTA was performed with *Escherichia coli* strain H1717 (kindly provided by Prof. Klaus Hantke, University of Tübingen, Germany), as described elsewhere^75^. *E. coli* strain H1717 carries the *lacZ* reporter gene under control of the Fur-regulated *fhuF* gene promoter. The promoter_*fhuF*_::*lacZ* gene fusion is exceptionally sensitive to changes of iron and Fur repressor concentrations. In the presence of iron, *E. coli* Fur repressor binds to the *fhuF* promoter region and the *lacZ* reporter gene is not expressed, rendering colorless *E. coli* H1717 colonies on MacConkey lactose agar plates (Lac^−^ phenotype). However, *E. coli* H1717 transformed with multicopy plasmids cloned with Fur binding sites will appear red on the plates (Lac^+^ phenotype), because the high number of newly introduced Fur boxes will cause the dissociation of the repressor Fur from the *fhuF* promoter, thus releasing the transcription of the *lacZ* gene.

The double-stranded DNA probes containing the sequences of the putative Fur boxes were cloned into high copy number pGEM^®^-T Easy vector (Promega). The resulting vectors were introduced into the *E. coli* strain H1717 and the transformants were plated onto MacConkey lactose agar containing 100 μg/mL amplicilin and under iron-rich condition. Plates were incubated for 18 h at 37 °C and the Lac phenotype was recorded. Circular pGEM^®^-T Easy vector without insert (i.e., not cloned with DNA probes) was used as a negative control, whereas vector cloned with the previously validated Fur box of the *K. pneumoniae entC* gene^40^ was used as a positive control.

### Expression and purification of *K. pneumoniae* Fur protein

The entire coding region of *fur* gene was PCR amplified from genomic DNA of *K. pneumoniae* with forward primer 5′-GTCGCATATGATGACTGACAACAATACC-3′, containing restriction site for *Nde*I (underlined nucleotides) and reverse primer 5′- TATCTCGAGTTATTTTTCCACCGCG-3′, containing restriction site for *Xho*I (underlined nucleotides). The PCR product was cloned into the expression vector pET-28a (Novagen) at the *Nde*I and *Xho*I sites, and the resulting plasmid was introduced into *E. coli* BL21(DE3).

To overexpress the *K. pneumoniae* histidine-tagged Fur protein (His-Fur), *E. coli* BL21(DE3) cells transformed with the recombinant plasmid were grown in LB medium to an O.D._600 nm_ of 0.4. At this point, isopropyl-β-D-thiogalactopyranoside (IPTG, Sigma-Aldrich) was added to a final concentration of 1 mM and the culture was incubated for 4 h. After IPTG induction, in-culture bacterial cell lysis was promoted by adding CelLytic^TM^ Express 1 mL Tablets (Sigma-Aldrich) to the culture, followed by incubation for 20 minutes at 37 °C and orbital shaker at 180 rpm. The His-Fur protein was then purified by affinity chromatography under native conditions by adding *HIS-Select Nickel Affinity Gel* (Sigma-Aldrich) to the lysed cell solution, according to the manufacturer’s protocol.

Eluted fractions containing the His-Fur were pooled, dialyzed overnight at 4 °C on storage buffer (20 mM Tris-HCl pH 7.8, 1 mM DTT, 0.1 mM MnSO4 and 10% glycerol v/v) and concentrated using *Pierce*^*®*^ *Concentrator* (Thermo Fisher Scientific) with molecular-mass cut off of 10 kDa, following the instructions of the manufacturer. The concentration of the purified His-Fur was determined by the Bradford method and the purity was verified by SDS-PAGE analysis.

### DNA Electrophoretic Mobility Shift Assay (EMSA)

The purified *K. pneumoniae* Fur protein and DNA fragments containing the putative Fur boxes were used on DNA Electrophoretic Mobility Shift Assays (EMSA). These DNA fragments were obtained by PCR amplifying the pGEM^®^-T Easy vectors cloned with the putative Fur boxes. Amplifications were done with universal M13 primers and the resulting 285 base pairs long fragments were then used as probes on EMSA. The negative control consisted of a 254 base pairs DNA fragment without Fur box sequence. This fragment was obtained by PCR amplifying a pGEM^®^-T Easy vector without insert (i.e., vector not cloned with the putative Fur boxes).

EMSA was performed as described elsewhere^73^, with minor modifications. In brief, 500 ηM of His-Fur protein was initially equilibrated for 10 minutes on ice in a 10 µL reaction volume containing 1x binding buffer (10 mM Tris, 50 mM KCl, 1 mM DTT, pH 7.5), 0.5 mM MgCl_2_, 0.5 mM MnSO_4_ and 2.5% (v/v) glycerol. To this binding reaction, 50 ηg of the DNA probes were added and the mixture was incubated for 20 minutes on ice. In addition, EMSA were carried out under divalent cation-free conditions by adding EDTA to a final concentration of 2 mM in the above reaction mixture.

Samples were loaded onto a 2% (w/v) agarose gel and submitted to electrophoresis for 30 minutes at 100 Volts in 1x Bis-Tris borate (pH 7.5) buffer containing 0.1 mM MnSO_4_. The agarose gels were stained after electrophoresis by soaking then on an ethidium bromide solution (0.5 ug/mL) for 15 minutes with gentle agitation. The DNA bands were visualized under UV light and recorded with a digital photodocumentation system (Gel Doc^TM^ XR, Biorad). Capture and analysis of the imagens were done with the Image Lab^TM^ Software version 5.0 (Biorad).

### Evaluation of usefulness of the candidate reference genes

To demonstrate the usefulness of the reference genes validated in this study, we selected the top two most stable reference genes, *recA* and *rho*, to normalize the relative expression levels of the Fur-regulated genes *cirA*, *iroN* and *fiu* in *K. pneumoniae* submitted to iron-replete and iron-limiting conditions.

*K. pneumoniae* cells were grown in LB medium until the exponential phase (O.D._600 nm_ = 0.6). At this point, ferrous iron (100 μM of FeSO_4_, final concentration) or an iron chelator (100 μM of 2,2′-Dipyridyl, final concentration) were added and after 30 minutes of incubation the cells were harvested by centrifugation. The control condition consisted of *K. pneumoniae* cells grown in LB medium and harvested at the exponential phase (O.D._600 nm_ = 0.6). All culture conditions were performed at least twice. The bacterial cell pellets were ressuspended in *RNAprotect*^*®*^ *Bacteria Reagent* for RNA stabilization and immediately submitted to total RNA extraction. The total RNA extraction and the first strand cDNA synthesis were conducted as above described.

The RT-qPCR reactions were carried out in triplicates in a 7300 Real-Time PCR System using the *Platinum*^*®*^ *SYBR*^*®*^ *Green qPCR SuperMix-UDG* in reaction mixture as previously described. Primers for *cirA*, *iroN* and *fiu* genes were designed with Primer3 *v. 0.4.0* using the entire coding region of the selected genes. The primers were designed in order to have about 20 bases of length, melting temperature of 60 °C, and amplicon size ranging from 95 to 105 base pair (see Supplementary Table S27). Before the Real-time PCR assays, primers for *cirA*, *iroN* and *fiu* genes were tested by conventional PCR followed by agarose gel electrophoresis (Supplementary Fig. S4). RT-qPCR data were normalized with the top two most stable reference genes, *recA* and *rho*, individually and with a normalization factor calculated as the geometric mean of the expression levels of the two genes. To emphasize the need of using suitable endogenous genes, *ffh* gene, which was considered the least stable gene, was also used in the normalization of the expression levels of the Fur-regulated genes. In addition, RT-qPCR data were normalized with *rrsH* gene, which encodes 16S ribosomal RNA subunit, since this gene is commonly used as endogenous control in RT-qPCR experiments. The relative expression levels of the target genes were calculated according to the comparative critical threshold (ΔΔC_T_) method^76^. GraphPad Prism 7.00 (GraphPad Software, Inc.) was used for the statistical analyses. The differences on the expression levels were evaluated by Student’s *t* test with correction for multiple tests (Holm-Sidak method). The differences were considered statistically significant with *p*-values ≤ 0.05.

### Data Availability

The datasets generated and analyzed during the current study are available from the corresponding author on reasonable request.

## Electronic supplementary material

Supplementary Information
