# Supplementary material for: Selection and validation of reference genes for gene expression studies in Klebsiella pneumoniae using Reverse Transcription Quantitative real-time PCR
Source: Sci Rep. 2018 Jun 13;8:9001. doi: 10.1038/s41598-018-27420-2 (PMC5998039; doi:10.1038/s41598-018-27420-2)
Supplement: Supplementary file 1 — Supplementary Information [file 41598_2018_27420_MOESM1_ESM.pdf]

SUPPLEMENTARY INFORMATION

Selection and validation of reference genes for gene expression studies in *Klebsiella pneumoniae* using Reverse Transcription Quantitative real-time PCR

Ana Érika Inácio Gomes[1], Leonardo Prado Stuchi[1], Nathália Maria Gonçalves Siqueira[1], João Batista Henrique[1], Renato Vicentini[2], Marcelo Lima Ribeiro[3], Michelle Darrieux[1], Lúcio Fábio Caldas Ferraz[1,*]

[1]Laboratório de Biologia Molecular de Microrganismos, Universidade São Francisco, Bragança Paulista, SP, 12916-900, Brasil

[2]Centro de Biologia Molecular e Engenharia Genética, Universidade Estadual de Campinas, Campinas, SP, 13083-875, Brasil

[3]Unidade Integrada de Farmacologia e Gastroenterologia (UNIFAG), Universidade São Francisco, Bragança Paulista, SP, 12916-900, Brasil

[*]Corresponding author: lucio.ferraz@usf.edu.br

SUPPLEMENTARY FIGURE

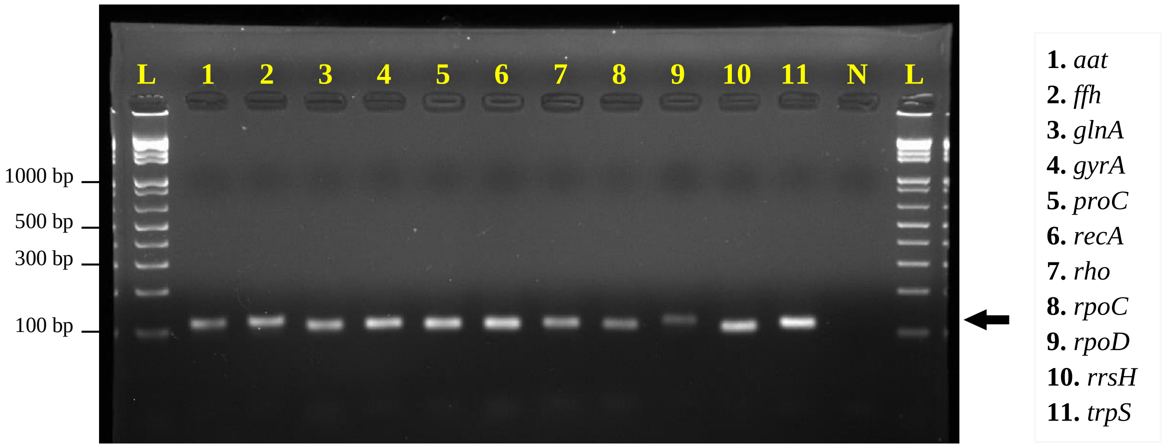

FIGURE S1. Agarose gel (1.5%) electrophoresis showing single PCR products of expected size (arrow), thus confirming the specificity of the pairs of primers for each gene. **L**, 1 Kb Plus DNA ladder (Thermo Fisher Scientific). **N**, negative control (PCR reaction without template DNA and with pair of primers for *trpS* gene).

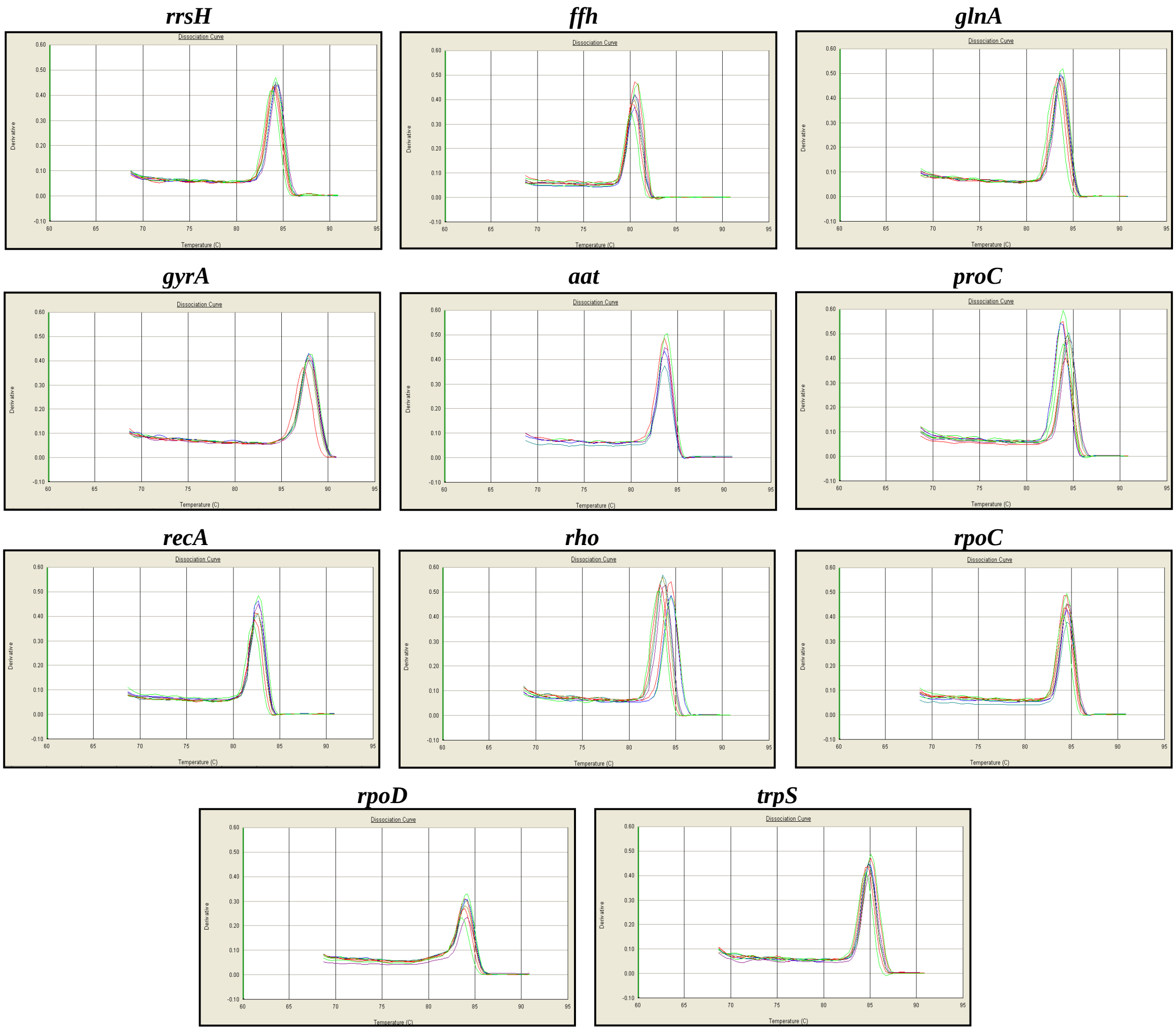

FIGURE S2. Dissociation-curve analyses showing only single peaks and the absence of primer-dimers formation and nonspecific amplification.

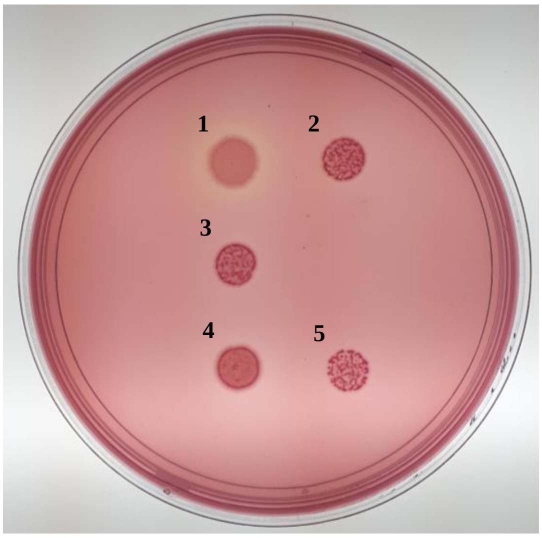

a)

1. **Lac** $^{-}$ (Furta-negative phenotype)
2. **Lac** $^{+}$ (Furta-positive phenotype)
3. ***cirA*.**
4. ***iroN*.**
5. ***fiu*.**

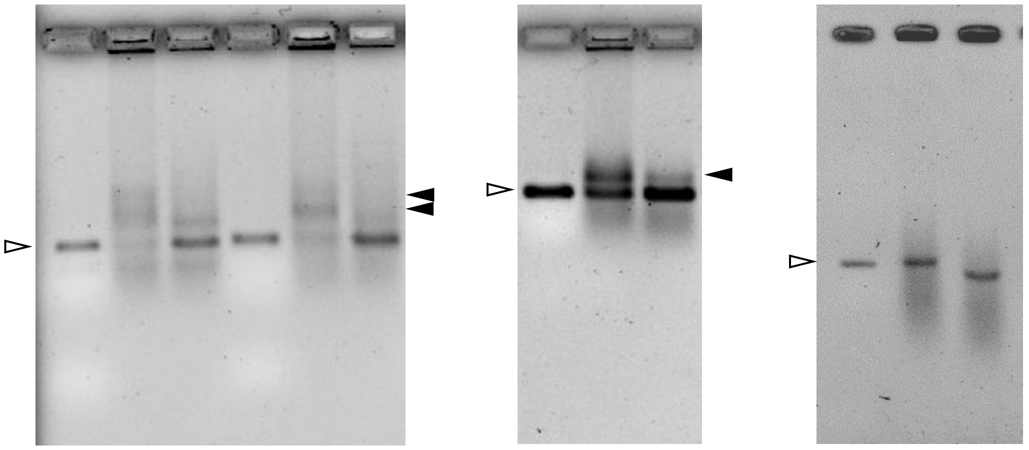

b)

FIGURE S3. Full-length figures showing the validation of the putative Fur boxes identified on the upstream region of the genes *cirA*, *iroN* and *fiu* by FURTA (**a**) and EMSA (**b**). In **a**) **FURTA:** Lac^{+} indicates FURTA-positive phenotype, whereas Lac^{-} indicates FURTA-negative phenotype. All putative Fur boxes resulted on red *E. coli* H1717 colonies on MacConkey plates, which were interpreted as FURTA positive results. **b) EMSA:** Lanes 1, 2 and 3 contained 50 ηg of the respective DNA probes. The DNA probes were incubated with 500 ηM of His-Fur protein either in the presence of divalent cation (Lanes 2) or under divalent cation-free conditions by adding 2 mM EDTA (Lanes 3). Open arrowheads indicate the free DNA probes, while closed arrowheads indicate the mobility shift corresponding to the Fur/DNA complexes.

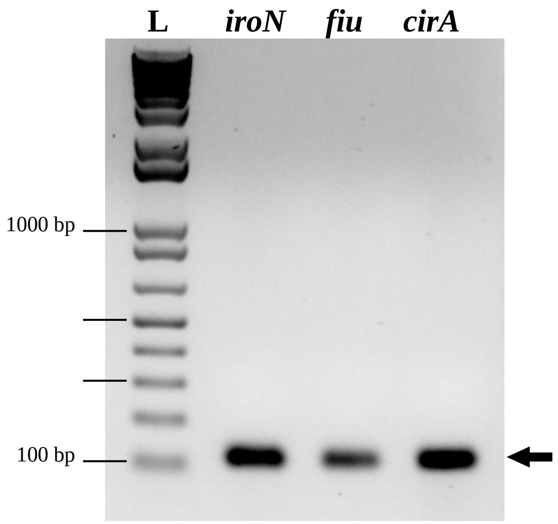

FIGURE S4. Agarose gel (1.5%) electrophoresis showing single PCR products of expected size (arrow) for *iroN*, *fiu* and *cirA* genes from *K. pneumoniae* strain ATCC 10031. **L**, 1 Kb Plus DNA ladder (Thermo Fisher Scientific).

SUPPLEMENTARY TABLES

TABLE S1. Functional category, locus number, product name and function of each selected reference gene candidates.

Functional categories	Gene/Locus	Product Name[1]	Function[2]
Cell metabolism	***proC*** (KPN_00329)	Pyrroline-5-carboxylate reductase	Catalyzes the formation of L-proline from pyrroline-5-carboxylate.
	glnA (KPN_04178)	Glutamine synthetase	Glutamine synthesis from ammonia and glutamate; also functions in the assimilation of ammonia.
DNA replication	***gyrA*** (KPN_02640)	DNA gyrase subunit A	ATP-dependent breakage, passage and rejoining of double-stranded DNA.
	recA (KPN_03031)	Recombinase A	Catalyzes the hydrolysis of ATP in the presence of single-stranded DNA, the ATP-dependent uptake of single-stranded DNA by duplex DNA, and the ATP-dependent hybridization of homologous single-stranded DNAs.
DNA transcription	***rpoD*** (KPN_03474)	RNA polymerase sigma factor RpoD	Sigma factors promote the attachment of RNA polymerase to specific initiation sites. RpoD is the primary sigma factor of bacteria.
	rho (KPN_04283)	Transcription termination factor Rho	An RNA-DNA helicase that actively releases nascent mRNAs from paused transcription complexes.
	rpoC (KPN_04366)	DNA-directed RNA polymerase subunit beta’	RNA polymerase subunit beta' binds to sigma factor allowing it to bind to the -10 region of the promoter.
Protein synthesis	***rrsH*** (KPN_00215)	16S ribosomal RNA subunit	Component of the small subunit (30S) of the ribosomes.
	aat (KPN_00917)	Leucyl/phenylalanyl tRNA protein transferase	It works in the N-end rule pathway; transfers Leu, Phe, Met, from aminoacyl-tRNAs to proteins containing an N-terminal arginine, lysine or histidine.
	ffh (KPN_02933)	Signal recognition particle protein	Forms a signal recognition particle with 4.5S RNA involved in targeting and integration of inner membrane proteins.
	trpS (KPN_04752)	Tryptophanyl-tRNA synthetase II	Catalyzes a two-step reaction, first charging a tryptophan molecule by linking its carboxyl group to the alpha-phosphate of ATP, followed by transfer of the aminoacyl-adenylate to its tRNA.

1) Product name according to the annotated genome sequence of *Klebsiella pneumoniae* MGH 78578 (GenBank accession number CP000647.1). **2)** Function according to NCBI Reference Sequence NC_009648.1 and Gene Ontology.

Table S2. Percentage sequence identity of *aat* gene among strains of *K. pneumoniae* from phylogroups KpI, KpII and KpIII.

***aat* gene**	**KpI**									**KpII**		**KpIII**
**Strain**	**1084**	**ATCC 10031**	**CG43**	**HS11286**	**JM45**	**KCTC 2242**	**Kp13**	**MGH 78578**	**NTUH-K2044**	**ATCC 700603**	**HKUOPA4**	**342**
**1084**	–	**99%** (698/702)	**99%** (698/702)	**99%** (696/697)	**99%** (701/702)	**99%** (699/702)	**99%** (701/702)	**99%** (698/702)	**100%** (702/702)	**95%** (666/699)	**95%** (666/699)	**91%** (634/697)
**ATCC 10031**		–	**100%** (702/702)	**99%** (694/697)	**99%** (699/702)	**99%** (701/702)	**99%** (699/702)	**99%** (700/702)	**99%** (698/702)	**95%** (662/699)	**95%** (662/699)	**91%** (634/697)
**CG43**			–	**99%** (694/697)	**99%** (699/702)	**99%** (701/702)	**99%** (699/702)	**99%** (700/702)	**99%** (698/702)	**95%** (662/699)	**95%** (662/699)	**91%** (634/697)
**HS11286**				–	**100%** (697/697)	**99%** (695/697)	**100%** (697/697)	**99%** (694/697)	**99%** (696/697)	**95%** (663/697)	**95%** (663/697)	**91%** (633/697)
**JM45**					–	**99%** (700/702)	**100%** (702/702)	**99%** (699/702)	**99%** (701/702)	**95%** (665/699)	**95%** (665/699)	**91%** (633/697)
**KCTC 2242**						–	**99%** (700/702)	**99%** (701/702)	**99%** (699/702)	**95%** (663/699)	**95%** (663/699)	**91%** (635/697)
**Kp13**							–	**99%** (699/702)	**99%** (701/702)	**95%** (665/699)	**95%** (665/699)	**91%** (633/697)
**MGH 78578**								–	**99%** (698/702)	**95%** (662/699)	**95%** (662/699)	**91%** (634/697)
**NTUH-K2044**									–	**95%** (666/699)	**95%** (665/699)	**91%** (634/697)
**ATCC 700603**										–	**99%** (696/705)	**93%** (653/705)
**HKUOPA4**											–	**92%** (652/705)
**342**												–

Table S3. Percentage sequence identity of *ffh* gene among strains of *K. pneumoniae* from phylogroups KpI, KpII and KpIII.

***ffh* gene**	**KpI**									**KpII**		**KpIII**
**Strain**	**1084**	**ATCC 10031**	**CG43**	**HS11286**	**JM45**	**KCTC 2242**	**Kp13**	**MGH 78578**	**NTUH-K2044**	**ATCC 700603**	**HKUOPA4**	**342**
**1084**	–	**99%** (1353/1365)	**99%** (1354/1365)	**99%** (1357/1365)	**99%** (1357/1365)	**99%** (1354/1365)	**99%** (1358/1365)	**99%** (1356/1365)	**100%** (1365/1365)	**97%** (1323/1365)	**97%** (1322/1365)	**98%** (1332/1365)
**ATCC 10031**		–	**99%** (1364/1365)	**99%** (1355/1365)	**99%** (1355/1365)	**99%** (1352/1365)	**99%** (1356/1365)	**99%** (1358/1365)	**99%** (1353/1365)	**97%** (1323/1365)	**97%** (1322/1365)	**98%** (1334/1365)
**CG43**			–	**99%** (1356/1365)	**99%** (1356/1365)	**99%** (1353/1365)	**99%** (1357/1365)	**99%** (1359/1365)	**99%** (1354/1365)	**97%** (1324/1365)	**97%** (1323/1365)	**98%** (1335/1365)
**HS11286**				–	**100%** (1365/1365)	**99%** (1354/1365)	**99%** (1362/1365)	**99%** (1360/1365)	**99%** (1357/1365)	**97%** (1323/1365)	**97%** (1322/1365)	**98%** (1332/1365)
**JM45**					–	**99%** (1354/1365)	**99%** (1362/1365)	**99%** (1360/1365)	**99%** (1357/1365)	**97%** (1323/1365)	**97%** (1322/1365)	**98%** (1332/1365)
**KCTC 2242**						–	**99%** (1355/1365)	**99%** (1355/1365)	**99%** (1354/1365)	**97%** (1322/1365)	**97%** (1319/1365)	**98%** (1333/1365)
**Kp13**							–	**99%** (1363/1365)	**99%** (1358/1365)	**97%** (1324/1365)	**97%** (1323/1365)	**98%** (1333/1365)
**MGH 78578**								–	**99%** (1356/1365)	**97%** (1322/1365)	**97%** (1321/1365)	**98%** (1333/1365)
**NTUH-K2044**									–	**97%** (1323/1365)	**97%** (1322/1365)	**98%** (1332/1365)
**ATCC 700603**										–	**99%** (1358/1365)	**97%** (1319/1365)
**HKUOPA4**											–	**96%** (1316/1365)
**342**												–

Table S4. Percentage sequence identity of *glnA* gene among strains of *K. pneumoniae* from phylogroups KpI, KpII and KpIII.

***glnA* gene**	**KpI**									**KpII**		**KpIII**
**Strain**	**1084**	**ATCC 10031**	**CG43**	**HS11286**	**JM45**	**KCTC 2242**	**Kp13**	**MGH 78578**	**NTUH-K2044**	**ATCC 700603**	**HKUOPA4**	**342**
**1084**	–	**99%** (1384/1386)	**99%** (1408/1410)	**100%** (1410/1410)	**100%** (1386/1386)	**99%** (1385/1386)	**99%** (1385/1386)	**99%** (1385/1386)	**100%** (1386/1386)	**98%** (1380/1410)	**98%** (1381/1410)	**98%** (1383/1410)
**ATCC 10031**		–	**100%** (1386/1386)	**99%** (1384/1386)	**99%** (1384/1386)	**99%** (1383/1386)	**99%** (1383/1386)	**99%** (1383/1386)	**99%** (1384/1386)	**98%** (1354/1386)	**98%** (1355/1386)	**98%** (1357/1386)
**CG43**			–	**99%** (1408/1410)	**99%** (1384/1386)	**99%** (1383/1386)	**99%** (1383/1386)	**99%** (1383/1386)	**99%** (1384/1386)	**98%** (1378/1410)	**98%** (1379/1410)	**98%** (1381/1410)
**HS11286**				–	**100%** (1386/1386)	**99%** (1385/1386)	**99%** (1385/1386)	**99%** (1385/1386)	**100%** (1386/1386)	**98%** (1380/1410)	**98%** (1381/1410)	**98%** (1383/1410)
**JM45**					–	**99%** (1385/1386)	**99%** (1385/1386)	**99%** (1385/1386)	**100%** (1386/1386)	**98%** (1356/1386)	**98%** (1357/1386)	**98%** (1359/1386)
**KCTC 2242**						–	**99%** (1384/1386)	**99%** (1384/1386)	**99%** (1385/1386)	**98%** (1355/1386)	**98%** (1356/1386)	**98%** (1358/1386)
**Kp13**							–	**100%** (1386/1386)	**99%** (1385/1386)	**98%** (1355/1386)	**98%** (1356/1386)	**98%** (1358/1386)
**MGH 78578**								–	**99%** (1385/1386)	**98%** (1355/1386)	**98%** (1356/1386)	**98%** (1358/1386)
**NTUH-K2044**									–	**98%** (1356/1386)	**98%** (1357/1386)	**98%** (1359/1386)
**ATCC 700603**										–	**99%** (1407/1410)	**99%** (1395/1410)
**HKUOPA4**											–	**99%** (1396/1410)
**342**												–

Table S5. Percentage sequence identity of *gyrA* gene among strains of *K. pneumoniae* from phylogroups KpI, KpII and KpIII.

***gyrA* gene**	**KpI**									**KpII**		**KpIII**
**Strain**	**1084**	**ATCC 10031**	**CG43**	**HS11286**	**JM45**	**KCTC 2242**	**Kp13**	**MGH 78578**	**NTUH-K2044**	**ATCC 700603**	**HKUOPA4**	**342**
**1084**	–	**99%** (2611/2626)	**99%** (2619/2634)	**99%** (2618/2634)	**99%** (2618/2634)	**99%** (2609/2626)	**99%** (2617/2634)	**99%** (2616/2634)	**99%** (2633/2634)	**97%** (2555/2634)	**97%** (2551/2634)	**97%** (2568/2634)
**ATCC 10031**		–	**100%** (2626/2626)	**99%** (2611/2626)	**99%** (2611/2626)	**99%** (2624/2626)	**99%** (2608/2626)	**99%** (2613/2626)	**99%** (2612/2626)	**97%** (2544/2626)	**97%** (2541/2626)	**97%** (2560/2626)
**CG43**			–	**99%** (2619/2634)	**99%** (2619/2634)	**99%** (2624/2626)	**99%** (2616/2634)	**99%** (2621/2634)	**99%** (2620/2634)	**97%** (2551/2634)	**97%** (2548/2634)	**97%** (2567/2634)
**HS11286**				–	**100%** (2634/2634)	**99%** (2611/2626)	**99%** (2629/2634)	**99%** (2619/2634)	**99%** (2619/2634)	**97%** (2550/2634)	**97%** (2547/2634)	**98%** (2570/2634)
**JM45**					–	**99%** (2611/2626)	**99%** (2629/2634)	**99%** (2619/2634)	**99%** (2619/2634)	**97%** (2550/2634)	**97%** (2547/2634)	**98%** (2570/2634)
**KCTC 2242**						–	**99%** (2609/2626)	**99%** (2613/2626)	**99%** (2610/2626)	97% (2542/2626)	**97%** (2539/2626)	**97%** (2558/2626)
**Kp13**							–	**99%** (2618/2634)	**99%** (2618/2634)	**97%** (2547/2634)	**97%** (2544/2634)	**97%** (2567/2634)
**MGH 78578**								–	**99%** (2617/2634)	**97%** (2549/2634)	**97%** (2545/2634)	**97%** (2566/2634)
**NTUH-K2044**									–	**97%** (2556/2634)	**97%** (2552/2634)	**98%** (2569/2634)
**ATCC 700603**										–	**99%** (2627/2634)	**96%** (2541/2634)
**HKUOPA4**											–	**96%** (2537/2634)
**342**												–

Table S6. Percentage sequence identity of *proC* gene among strains of *K. pneumoniae* from phylogroups KpI, KpII and KpIII.

***proC* gene**	**KpI**									**KpII**		**KpIII**
**Strain**	**1084**	**ATCC 10031**	**CG43**	**HS11286**	**JM45**	**KCTC 2242**	**Kp13**	**MGH 78578**	**NTUH-K2044**	**ATCC 700603**	**HKUOPA4**	**342**
**1084**	–	**99%** (808/810)	**99%** (808/810)	**99%** (808/810)	**99%** (808/810)	**99%** (806/810)	**99%** (807/810)	**99%** (808/810)	**100%** (810/810)	**93%** (756/810)	**93%** (757/810)	**94%** (762/810)
**ATCC 10031**		–	**100%** (810/810)	**100%** (810/810)	**100%** (816/816)	**99%** (810/816)	**99%** (813/816)	**100%** (816/816)	**99%** (808/810)	**94%** (758/810)	**94%** (759/810)	**94%** (764/810)
**CG43**			–	**100%** (810/810)	**100%** (810/810)	**99%** (804/810)	**99%** (807/810)	**100%** (810/810)	**99%** (808/810)	**94%** (758/810)	**94%** (759/810)	**94%** (764/810)
**HS11286**				–	**100%** (810/810)	**99%** (804/810)	**99%** (807/810)	**100%** (810/810)	**99%** (808/810)	**94%** (758/810)	**94%** (759/810)	**94%** (764/810)
**JM45**					–	**99%** (810/816)	**99%** (813/816)	**100%** (816/816)	**99%** (808/810)	**94%** (758/810)	**94%** (759/810)	**94%** (764/810)
**KCTC 2242**						–	99% (813/816)	**99%** (810/816)	**99%** (806/810)	**93%** (754/810)	**93%** (755/810)	**94%** (760/810)
**Kp13**							–	**99%** (813/816)	**99%** (807/810)	**93%** (757/810)	**94%** (758/810)	**94%** (763/810)
**MGH 78578**								–	**99%** (808/810)	**94%** (758/810)	**94%** (759/810)	**94%** (764/810)
**NTUH-K2044**									–	**93%** (756/810)	**93%** (757/810)	**94%** (762/810)
**ATCC 700603**										–	**99%** (801/810)	**94%** (765/810)
**HKUOPA4**											–	**94%** (762/810)
**342**												–

Table S7. Percentage sequence identity of *recA* gene among strains of *K. pneumoniae* from phylogroups KpI, KpII and KpIII.

***recA* gene**	**KpI**									**KpII**		**KpIII**
**Strain**	**1084**	**ATCC 10031**	**CG43**	**HS11286**	**JM45**	**KCTC 2242**	**Kp13**	**MGH 78578**	**NTUH-K2044**	**ATCC 700603**	**HKUOPA4**	**342**
**1084**	–	**99%** (1054/1059)	**99%** (1054/1059)	**99%** (1054/1059)	**99%** (1041/1050)	**99%** (1052/1059)	**99%** (1051/1059)	**99%** (1053/1059)	**100%** (1059/1059)	**95%** (1003/1059)	**95%** (1003/1059)	**95%** (1001/1059)
**ATCC 10031**		–	**100%** (1059/1059)	**99%** (1053/1059)	**99%** (1044/1050)	**99%** (1057/1059)	**99%** (1054/1059)	**99%** (1054/1059)	**99%** (1054/1059)	**95%** (1006/1059)	**95%** (1006/1059)	**95%** (1002/1059)
**CG43**			–	**99%** (1053/1059)	**99%** (1044/1050)	**99%** (1057/1059)	**99%** (1054/1059)	**99%** (1054/1059)	**99%** (1054/1059)	**95%** (1006/1059)	**95%** (1006/1059)	**95%** (1002/1059)
**HS11286**				–	**100%** (1050/1050)	**99%** (1055/1059)	**99%** (1054/1059)	**99%** (1054/1059)	**99%** (1050/1059)	**95%** (1010/1059)	**95%** (1010/1059)	**95%** (1002/1059)
**JM45**					–	**99%** (1046/1050)	**99%** (1045/1050)	**99%** (1046/1050)	**99%** (1041/1050)	**95%** (1001/1049)	**95%** (1001/1049)	**95%** (993/1049)
**KCTC 2242**						–	**99%** (1054/1059)	**99%** (1052/1059)	**99%** (1052/1059)	**95%** (1008/1059)	**95%** (1008/1059)	**94%** (1004/1059)
**Kp13**							–	**99%** (1053/1059)	**99%** (1051/1059)	**95%** (1009/1059)	**95%** (1009/1059)	**95%** (1003/1059)
**MGH 78578**								–	**99%** (1053/1059)	**95%** (1005/1059)	**95%** (1005/1059)	**95%** (1005/1059)
**NTUH-K2044**									–	**95%** (1003/1059)	**95%** (1003/1059)	**95%** (1001/1059)
**ATCC 700603**										–	**100%** (1059/1059)	**97%** (1032/1059)
**HKUOPA4**											–	**97%** (1032/1059)
**342**												–

Table S8. Percentage sequence identity of *rho* gene among strains of *K. pneumoniae* from phylogroups KpI, KpII and KpIII.

***rho* gene**	**KpI**									**KpII**		**KpIII**
**Strain**	**1084**	**ATCC 10031**	**CG43**	**HS11286**	**JM45**	**KCTC 2242**	**Kp13**	**MGH 78578**	**NTUH-K2044**	**ATCC 700603**	**HKUOPA4**	**342**
**1084**	–	**99%** (1257/1260)	**99%** (1257/1260)	**99%** (1257/1260)	**99%** (1257/1260)	**99%** (1257/1260)	**99%** (1256/1260)	**99%** (1255/1260)	**100%** (1260/1260)	**97%** (1218/1260)	**97%** (1218/1260)	**97%** (1217/1260)
**ATCC 10031**		–	**100%** (1260/1260)	**99%** (1258/1260)	**99%** (1258/1260)	**100%** (1260/1260)	**99%** (1259/1260)	**99%** (1258/1260)	**99%** (1257/1260)	**97%** (1219/1260)	**97%** (1219/1260)	**97%** (1218/1260)
**CG43**			–	**99%** (1258/1260)	**99%** (1258/1260)	**100%** (1260/1260)	**99%** (1259/1260)	**99%** (1258/1260)	**99%** (1257/1260)	**97%** (1219/1260)	**97%** (1219/1260)	**97%** (1218/1260)
**HS11286**				–	**100%** (1260/1260)	**99%** (1258/1260)	**99%** (1257/1260)	**99%** (1256/1260)	**99%** (1257/1260)	**97%** (1217/1260)	**97%** (1217/1260)	**97%** (1216/1260)
**JM45**					–	**99%** (1258/1260)	**99%** (1257/1260)	**99%** (1256/1260)	**99%** (1257/1260)	**97%** (1217/1260)	**97%** (1217/1260)	**97%** (1216/1260)
**KCTC 2242**						–	**99%** (1259/1260)	**99%** (1258/1260)	**99%** (1257/1260)	**97%** (1219/1260)	**97%** (1219/1260)	**97%** (1218/1260)
**Kp13**							–	**99%** (1257/1260)	**99%** (1256/1260)	**97%** (1218/1260)	**97%** (1218/1260)	**97%** (1217/1260)
**MGH 78578**								–	**99%** (1256/1260)	**97%** (1217/1260)	**97%** (1217/1260)	**97%** (1216/1260)
**NTUH-K2044**									–	**97%** (1218/1260)	**97%** (1218/1260)	**97%** (1217/1260)
**ATCC 700603**										–	**99%** (1258/1260)	**96%** (1213/1260)
**HKUOPA4**											–	**96%** (1213/1260)
**342**												–

Table S9. Percentage sequence identity of *rpoC* gene among strains of *K. pneumoniae* from phylogroups KpI, KpII and KpIII.

***rpoC* gene**	**KpI**									**KpII**		**KpIII**
**Strain**	**1084**	**ATCC 10031**	**CG43**	**HS11286**	**JM45**	**KCTC 2242**	**Kp13**	**MGH 78578**	**NTUH-K2044**	**ATCC 700603**	**HKUOPA4**	**342**
**1084**	–	**99%** (4218/4224)	**99%** (4218/4224)	**99%** (4214/4224)	**99%** (4214/4224)	**99%** (4213/4224)	**99%** (4217/4224)	**99%** (4219/4224)	**99%** (4223/4224)	**98%** (4155/4224)	**98%** (4156/4224)	**96%** (4054/4224)
**ATCC 10031**		–	**100%** (4224/4224)	**99%** (4218/4224)	**99%** (4218/4224)	**99%** (4217/4224)	**99%** (4221/4224)	**99%** (4221/4224)	**99%** (4219/4224)	**98%** (4159/4224)	**98%** (4160/4224)	**96%** (4060/4224)
**CG43**			–	**99%** (4218/4224)	**99%** (4218/4224)	**99%** (4217/4224)	**99%** (4221/4224)	**99%** (4221/4224)	**99%** (4219/4224)	**98%** (4159/4224)	**98%** (4160/4224)	**96%** (4060/4224)
**HS11286**				–	**100%** (4224/4224)	**99%** (4215/4224)	**99%** (4219/4224)	**99%** (4215/4224)	**99%** (4215/4224)	**98%** (4157/4224)	**98%** (4158/4224)	**96%** (4056/4224)
**JM45**					–	**99%** (4215/4224)	**99%** (4219/4224)	**99%** (4215/4224)	**99%** (4215/4224)	**98%** (4157/4224)	**98%** (4158/4224)	**96%** (4056/4224)
**KCTC 2242**						–	**99%** (4214/4224)	**99%** (4216/4224)	**99%** (4214/4224)	**98%** (4154/4224)	**98%** (4155/4224)	**96%** (4054/4224)
**Kp13**							–	**99%** (4218/4224)	**99%** (4218/4224)	**98%** (4158/4224)	**98%** (4159/4224)	**96%** (4057/4224)
**MGH 78578**								–	**99%** (4220/4224)	**98%** (4156/4224)	**98%** (4157/4224)	**96%** (4057/4224)
**NTUH-K2044**									–	**98%** (4156/4224)	**98%** (4157/4224)	**96%** (4055/4224)
**ATCC 700603**										–	**99%** (4218/4224)	**96%** (4068/4224)
**HKUOPA4**											–	**96%** (4065/4224)
**342**												–

Table S10. Percentage sequence identity of *rpoD* gene among strains of *K. pneumoniae* from phylogroups KpI, KpII and KpIII.

***rpoD* gene**	**KpI**									**KpII**		**KpIII**
**Strain**	**1084**	**ATCC 10031**	**CG43**	**HS11286**	**JM45**	**KCTC 2242**	**Kp13**	**MGH 78578**	**NTUH-K2044**	**ATCC 700603**	**HKUOPA4**	**342**
**1084**	–	**99%** (1839/1842)	**99%** (1839/1842)	**99%** (1836/1842)	**99%** (1836/1842)	**99%** (1836/1842)	**99%** (1836/1842)	**99%** (1840/1842)	**100%** (1842/1842)	**98%** (1802/1842)	**98%** (1799/1842)	**98%** (1796/1842)
**ATCC 10031**		–	**100%** (1842/1842)	**99%** (1839/1842)	**99%** (1839/1842)	**99%** (1837/1842)	**99%** (1839/1842)	**99%** (1841/1842)	**99%** (1839/1842)	**98%** (1805/1842)	**98%** (1802/1842)	**98%** (1799/1842)
**CG43**			–	**99%** (1839/1842)	**99%** (1839/1842)	**99%** (1837/1842)	**99%** (1839/1842)	**99%** (1841/1842)	**99%** (1839/1842)	**98%** (1805/1842)	**98%** (1802/1842)	**98%** (1799/1842)
**HS11286**				–	**100%** (1842/1842)	**99%** (1840/1842)	**99%** (1840/1842)	**99%** (1838/1842)	**99%** (1836/1842)	**98%** (1804/1842)	**98%** (1803/1842)	**98%** (1800/1842)
**JM45**					–	**99%** (1840/1842)	**99%** (1840/1842)	**99%** (1838/1842)	**99%** (1836/1842)	**98%** (1804/1842)	**98%** (1803/1842)	**98%** (1800/1842)
**KCTC 2242**						–	**99%** (1838/1842)	**99%** (1838/1842)	**99%** (1836/1842)	**98%** (1802/1842)	**98%** (1801/1842)	**98%** (1798/1842)
**Kp13**							–	**99%** (1838/1842)	**99%** (1836/1842)	**98%** (1804/1842)	**98%** (1803/1842)	**98%** (1800/1842)
**MGH 78578**								–	**99%** (1840/1842)	**98%** (1804/1842)	**98%** (1801/1842)	**98%** (1798/1842)
**NTUH-K2044**									–	**98%** (1802/1842)	**98%** (1799/1842)	**98%** (1796/1842)
**ATCC 700603**										–	99% (1835/1842)	**98%** (1806/1842)
**HKUOPA4**											–	**98%** (1809/1842)
**342**												–

Table S11. Percentage sequence identity of *rrsH* gene among strains of *K. pneumoniae* from phylogroups KpI, KpII and KpIII.

***rrsH* gene**	**KpI**									**KpII**		**KpIII**
**Strain**	**1084**	**ATCC 10031**	**CG43**	**HS11286**	**JM45**	**KCTC 2242**	**Kp13**	**MGH 78578**	**NTUH-K2044**	**ATCC 700603**	**HKUOPA4**	**342**
**1084**	–	**99%** (1538/1544)	**99%** (1542/1544)	**99%** (1541/1544)	**99%** (1538/1544)	**99%** (1542/1544)	**99%** (1537/1544)	**99%** (1538/1544)	**99%** (1541/1544)	**99%** (1535/1544)	**99%** (1536/1544)	**99%** (1530/1544)
**ATCC 10031**		–	**99%** (1539/1544)	**99%** (1538/1544)	**99%** (1533/1544)	**99%** (1539/1544)	**99%** (1534/1544)	**99%** (1535/1544)	**99%** (1538/1544)	**99%** (1534/1544)	**99%** (1535/1544)	**99%** (1536/1544)
**CG43**			–	**99%** (1540/1544)	**99%** (1538/1544)	**99%** (1541/1544)	**99%** (1539/1544)	**99%** (1540/1544)	**99%** (1543/1544)	**99%** (1546/1554)	**99%** (1547/1554)	**99%** (1541/1544)
**HS11286**				–	**99%** (1538/1544)	**99%** (1542/1544)	**99%** (1535/1544)	**99%** (1536/1544)	**99%** (1541/1544)	**99%** (1535/1544)	**99%** (1538/1544)	**99%** (1532/1544)
**JM45**					–	**99%** (1537/1544)	**99%** (1541/1544)	**99%** (1542/1544)	**99%** (1539/1544)	**99%** (1538/1544)	**99%** (1535/1544)	**99%** (1535/1544)
**KCTC 2242**						–	**99%** (1536/1544)	**99%** (1537/1544)	**99%** (1540/1544)	**99%** (1537/1544)	**99%** (1538/1544)	**99%** (1531/1544)
**Kp13**							–	**99%** (1543/1544)	**99%** (1538/1544)	**99%** (1539/1544)	**99%** (1534/1544)	**99%** (1534/1544)
**MGH 78578**								–	**99%** (1539/1544)	**99%** (1540/1544)	**99%** (1535/1544)	**99%** (1535/1544)
**NTUH-K2044**									–	**99%** (1535/1544)	**99%** (1538/1544)	**99%** (1532/1544)
**ATCC 700603**										–	**99%** (1549/1554)	**99%** (1544/1544)
**HKUOPA4**											–	**99%** (1541/1544)
**342**												–

Table S12. Percentage sequence identity of *trpS* gene among strains of *K. pneumoniae* from phylogroups KpI, KpII and KpIII.

***trpS* gene**	**KpI**									**KpII**		**KpIII**
**Strain**	**1084**	**ATCC 10031**	**CG43**	**HS11286**	**JM45**	**KCTC 2242**	**Kp13**	**MGH 78578**	**NTUH-K2044**	**ATCC 700603**	**HKUOPA4**	**342**
**1084**	–	**99%** (994/1008)	**99%** (994/1008)	**99%** (994/1008)	**99%** (994/1008)	**99%** (1000/1008)	**99%** (993/1008)	**98%** (987/1008)	**100%** (1008/1008)	**94%** (947/1008)	**94%** (945/1008)	**95%** (959/1008)
**ATCC 10031**		–	**100%** (1008/1008)	**99%** (996/1008)	**99%** (996/1008)	**99%** (1002/1008)	**99%** (995/1008)	**98%** (989/1008)	**99%** (994/1008)	**94%** (947/1008)	**94%** (945/1008)	**94%** (949/1008)
**CG43**			–	**99%** (996/1008)	**99%** (996/1008)	**99%** (1002/1008)	**99%** (995/1008)	**98%** (989/1008)	**99%** (994/1008)	**94%** (947/1008)	**94%** (945/1008)	**94%** (949/1008)
**HS11286**				–	**100%** (1008/1008)	**99%** (1002/1008)	**99%** (999/1008)	**98%** (989/1008)	**99%** (994/1008)	**94%** (943/1008)	**93%** (941/1008)	**94%** (951/1008)
**JM45**					–	**99%** (1002/1008)	**99%** (999/1008)	**98%** (989/1008)	**99%** (994/1008)	**94%** (943/1008)	**93%** (941/1008)	**94%** (951/1008)
**KCTC 2242**						–	**99%** (1001/1008)	**99%** (995/1008)	**99%** (1000/1008)	**94%** (945/1008)	**94%** (943/1008)	**95%** (953/1008)
**Kp13**							–	**98%** (988/1008)	**99%** (993/1008)	**94%** (946/1008)	**94%** (944/1008)	**95%** (956/1008)
**MGH 78578**								–	**98%** (987/1008)	**94%** (946/1008)	**94%** (944/1008)	**95%** (954/1008)
**NTUH-K2044**									–	**94%** (947/1008)	**94%** (945/1008)	**95%** (959/1008)
**ATCC 700603**										–	**99%** (1002/1008)	**94%** (952/1008)
**HKUOPA4**											–	**94%** (950/1008)
**342**												–

TABLE S13. Statistical analyses by BestKeeper.

Genes	**GM** [C_T][1]	**SD** [± C_T][2]	**CV** [% C_T][3]
aat *	38.79	1.56	4.03
ffh	23.90	1.00	4.49
glnA	23.76	0.95	4.01
gyrA	24.45	0.84	3.45
proC	24.91	0.59	2.37
recA	22.40	0.47	2.10
rho	23.91	0.69	2.90
rpoC	23.26	0.83	3.59
rpoD	25.04	0.74	2.97
rrsH	10.58	0.43	4.10
trpS *	29.09	1.50	5.14

1. Geometric mean of the C_T values; **2.** Standard deviation of the C_{Ts}; **3.** Coefficient of variation of the C_{Ts}; ***** Genes excluded from the subsequent statistical analyses

TABLE S14. Comparison of the BestKeeper, NormFinder and geNorm results calculated by RefFinder with those calculated by their corresponding original softwares. The superscripted numbers in parentheses (boldface and underlined) indicate the ranking of the genes with the most stable expression according to each analysis.

	**BestKeeper**		**NormFinder**		**GeNorm**	
Gene	Original software	RefFinder analysis	Original software	RefFinder analysis	Original software	RefFinder analysis
recA	0.849 **(1)**	0.849 **(1)**	0.296 **(1)**	0.306 **(1)**	0.67 **(5)**	0.764 **(5)**
rho	0.816 **(2)**	0.816 **(2)**	0.328 **(2)**	0.493 **(2)**	0.78 **(6)**	0.85 **(6)**
glnA	0.814 **(3)**	0.814 **(3)**	0.776 (9)	0.902 (7)	0.92 **(8)**	0.979 **(8)**
gyrA	0.739 **(4)**	0.739 **(4)**	0.535 (5)	0.777 (6)	0.86 **(7)**	0.921 **(7)**
ffh	0.712 **(5)**	0.712 **(5)**	0.760 (8)	1.092 (9)	0.99 **(9)**	1.035 **(9)**
rpoD	0.687 **(6)**	0.687 **(6)**	0.674 (7)	0.727 (4)	0.49 (3)	0.592 **(1/2)**
proC	0.66 **(7)**	0.66 **(7)**	0.423 **(3)**	0.632 **(3)**	0.44 (1/2)	0.617 (3)
rpoC	0.541 **(8)**	0.541 **(8)**	0.614 (6)	0.932 (8)	0.44 **(1/2)**	0.592 **(1/2)**
rrsH	0.42 **(9)**	0.42 **(9)**	0.515 (4)	0.765 (5)	0.60 **(4)**	0.724 **(4)**

TABLE S15. Primer sequences of the proposed reference genes (and corresponding locus tag) for *Klebsiella pneumoniae subsp. pneumoniae* 1084, phylogroup KpI (RefSeq: NC_018522.1).

Gene	Locus tag	Forward (F) and Reverse (R) primer sequences (5' > 3')	Amplicon size
proC	A79E_3956	F- GATTGCCGATATCGTCTTCG<br>R- GAGACCACCAGCGACTCTTT	99 bp
recA	A79E_1066	F- TTAAACAGGCCGAATTCCAG<br>R- CCGCTTTCTCAATCAGCTTC	99 bp
rho	A79E_4905	F- AACTACGACAAGCCGGAAAA<br>R- ACCGTTACCACGCTCCATAC	99 bp
rpoD	A79E_0644	F- TCCGGTGCATATGATTGAGA<br>R- ATACGCTCAGCCAGCTCTTC	105 bp

TABLE S16. Primer sequences of the proposed reference genes (and corresponding locus tag) for *Klebsiella pneumoniae* ATCC 10031, phylogroup KpI (ERS747594).

Gene	Locus tag	Forward (F) and Reverse (R) primer sequences (5' > 3')	Amplicon size
proC	NCTC7427_03519	F- GATTGCCGATATCGTCTTCG<br>R- GAGACCACCAGCGACTCTTT	99 bp
recA	NCTC7427_04077	F- TTAAACAGGCCGAATTCCAG<br>R- CCGCTTTCTCAATCAGCTTC	99 bp
rho	NCTC7427_04869	F- AACTACGACAAGCCGGAAAA<br>R- ACCGTTACCACGCTCCATAC	99 bp
rpoD	NCTC7427_04564	F- TCCGGTGCATATGATTGAGA<br>R- ATACGCTCAGCCAGCTCTTC	105 bp

TABLE S17. Primer sequences of the proposed reference genes (and corresponding locus tag) for *Klebsiella pneumoniae* CG43, phylogroup KpI (RefSeq: NC_022566.1).

Gene	Locus tag	Forward (F) and Reverse (R) primer sequences (5' > 3')	Amplicon size
proC	D364_RS01715	F- GATTGCCGATATCGTCTTCG<br>R- GAGACCACCAGCGACTCTTT	99 bp
recA	D364_RS15830	F- TTAAACAGGCCGAATTCCAG<br>R- CCGCTTTCTCAATCAGCTTC	99 bp
rho	D364_RS22365	F- AACTACGACAAGCCGGAAAA<br>R- ACCGTTACCACGCTCCATAC	99 bp
rpoD	D364_RS18330	F- TCCGGTGCATATGATTGAGA<br>R- ATACGCTCAGCCAGCTCTTC	105 bp

TABLE S18. Primer sequences of the proposed reference genes (and corresponding locus tag) for *Klebsiella pneumoniae subsp. pneumoniae* HS11286, phylogroup KpI (RefSeq: NC_016845.1).

**Gene**	**Locus tag**	**Forward (F) and Reverse (R) primer sequences (5’ > 3’)**	**Amplicon size**
proC	KPHS_10670	F- GATTGCCGATATCGTCTTCG<br>R- GAGACCACCAGCGACTCTTT	99 bp
recA	KPHS_41020	F- TTAAACAGGCCGAATTCCAG<br>R- CCGCTTTCTCAATCAGCTTC	99 bp
rho	KPHS_01300	F- AACTACGACAAGCCGGAAAA<br>R- ACCGTTACCACGCTCCATAC	99 bp
rpoD	KPHS_46060	F- TCCGGTGCATATGATTGAGA<br>R- ATACGCTCAGCCAGCTCTTC	105 bp

TABLE S19. Primer sequences of the proposed reference genes (and corresponding locus tag) for *Klebsiella pneumoniae* JM45, phylogroup KpI (RefSeq: NC_022082.1).

**Gene**	**Locus tag**	**Forward (F) and Reverse (R) primer sequences (5’ > 3’)**	**Amplicon size**
proC	N559_4083	F- GATTGCCGATATCGTCTTCG<br>R- GAGACCACCAGCGACTCTTT	99 bp
recA	N559_1206	F- TTAAACAGGCCGAATTCCAG<br>R- CCGCTTTCTCAATCAGCTTC	99 bp
rho	N559_5002	F- AACTACGACAAGCCGGAAAA<br>R- ACCGTTACCACGCTCCATAC	99 bp
rpoD	N559_0700	F- TCCGGTGCATATGATTGAGA<br>R- ATACGCTCAGCCAGCTCTTC	105 bp

TABLE S20. Primer sequences of the proposed reference genes (and corresponding locus tag) for *Klebsiella pneumoniae* KCTC 2242, phylogroup KpI (RefSeq: NC_017540.1).

**Gene**	**Locus tag**	**Forward (F) and Reverse (R) primer sequences (5’ > 3’)**	**Amplicon size**
proC	KPN2242_RS04295	F- GATTGCCGATATCGTCTTCG<br>R- GAGACCACCAGCGACTCTTT	99 bp
recA	KPN2242_RS18970	F- TTAAACAGGCCGAATTCCAG<br>R- CCGCTTTCTCAATCAGCTTC	99 bp
rho	KPN2242_RS25525	F- AACTACGACAAGCCGGAAAA<br>R- ACCGTTACCACGCTCCATAC	99 bp
rpoD	KPN2242_RS21380	F- TCCGGTGCATATGATTGAGA<br>R- ATACGCTCAGCCAGCTCTTC	105 bp

TABLE S21. Primer sequences of the proposed reference genes (and corresponding locus tag) for *Klebsiella pneumoniae subsp. pneumoniae* Kp13, phylogroup KpI (RefSeq: NZ_003999.1).

Gene	Locus tag	Forward (F) and Reverse (R) primer sequences (5' > 3')	Amplicon size
proC	KP13_02087	F- GATTGCCGATATCGTCTTCG<br>R- GAGACCACCAGCGACTCTTT	99 bp
recA	KP13_02602	F- TTAAACAGGCCGAATTCCAG<br>R- CCGCTTTCTCAATCAGCTTC	99 bp
rho	KP13_32218	F- AACTACGACAAGCCGGAAAA<br>R- ACCGTTACCACGCTCCATAC	99 bp
rpoD	KP13_31791	F- TCCGGTGCATATGATTGAGA<br>R- ATACGCTCAGCCAGCTCTTC	105 bp

TABLE S22. Primer sequences of the proposed reference genes (and corresponding locus tag) for *Klebsiella pneumoniae subsp. pneumoniae* NTUH-K2044, phylogroup KpI (RefSeq: NC_012731.1).

Gene	Locus tag	Forward (F) and Reverse (R) primer sequences (5' > 3')	Amplicon size
proC	KP1_RS05650	F- GATTGCCGATATCGTCTTCG<br>R- GAGACCACCAGCGACTCTTT	99 bp
recA	KP1_RS20055	F- TTAAACAGGCCGAATTCCAG<br>R- CCGCTTTCTCAATCAGCTTC	99 bp
rho	KP1_RS00705	F- AACTACGACAAGCCGGAAAA<br>R- ACCGTTACCACGCTCCATAC	99 bp
rpoD	KP1_RS22225	F- TCCGGTGCATATGATTGAGA<br>R- ATACGCTCAGCCAGCTCTTC	105 bp

TABLE S23. Primer sequences of the proposed reference genes (and corresponding locus tag) for *Klebsiella pneumoniae subsp. pneumoniae* MGH 78578, phylogroup KpI (RefSeq: NC_009648.1).

Gene	Locus tag	Forward (F) and Reverse (R) primer sequences (5' > 3')	Amplicon size
proC	KPN_RS01765	F- GATTGCCGATATCGTCTTCG<br>R- GAGACCACCAGCGACTCTTT	99 bp
recA	KPN_RS16255	F- TTAAACAGGCCGAATTCCAG<br>R- CCGCTTTCTCAATCAGCTTC	99 bp
rho	KPN_RS23075	F- AACTACGACAAGCCGGAAAA<br>R- ACCGTTACCACGCTCCATAC	99 bp
rpoD	KPN_RS18620	F- TCCGGTGCATATGATTGAGA<br>R- ATACGCTCAGCCAGCTCTTC	105 bp

TABLE S24. Primer sequences of the proposed reference genes (and corresponding locus tag) for *Klebsiella quasipneumoniae* strain HKUOPA4, phylogroup KpII (RefSeq: NZ_CP014154.1). Nucleotides different from the primers of the KpI strains are in bold and underlined.

Gene	Locus tag	Forward (F) and Reverse (R) primer sequences (5' > 3')	Amplicon size
proC	AWV58_RS00665	F- **A**ATTGCCGATATCGTCTTCG<br>R- GAGACCACCAGCGACTCTTT	99 bp
recA	AWV58_RS14870	F- T**C**AAACAGGC**T**GAATTCCAG<br>R- CCGCTTTCTCAATCAGCTTC	99 bp
rho	AWV58_RS21150	F- AACTACGACAAGCCGGAAAA<br>R- ACCGTTACCACG**C**TCCATAC	99 bp
rpoD	AWV58_RS17120	F- TCCGGTGCATATGATTGAGA<br>R- AT**G**CGCTC**G**GCCAGCTCTTC	105 bp

TABLE S25. Primer sequences of the proposed reference genes (and corresponding locus tag) for *Klebsiella quasipneumoniae* strain ATCC 700603, phylogroup KpII (RefSeq: NZ_CP014696.2). Nucleotides different from the primers of the KpI strains are in bold and underlined.

Gene	Locus tag	Forward (F) and Reverse (R) primer sequences (5' > 3')	Amplicon size
proC	AVR78_RS25190	F- **A**ATTGCCGATATCGTCTTCG<br>R- GAGACCACCAGCGACTCTTT	99 bp
recA	AVR78_RS13765	F- T**C**AAACAGGC**T**GAATTCCAG<br>R- CCGCTTTCTCAATCAGCTTC	99 bp
rho	AVR78_RS20240	F- AACTACGACAAGCCGGAAAA<br>R- ACCGTTACCACG**C**TCCATAC	99 bp
rpoD	AVR78_RS16040	F- TCCGGTGCATATGATTGAGA<br>R- AT**G**CGCTCAGCCAGCTCTTC	105 bp

TABLE S26. Primer sequences of the proposed reference genes (and corresponding locus tag) for *Klebsiella varicella* (formerly identified as *K. pneumoniae* strain 342), phylogroup KpIII (RefSeq: NC_011283.1). Nucleotides different from the primers of the KpI strains are in bold and underlined.

Gene	Locus tag	Forward (F) and Reverse (R) primer sequences (5' > 3')	Amplicon size
proC	KPK_RS20665	F- GATTGCCGATATCGTCTTCG<br>R- GAGACCACCAGCGACTCTTT	99 bp
recA	KPK_RS05310	F- T**C**AAACAGGC**T**GAATTCCAG<br>R- CCGCTTTCTCAATCAGCTTC	99 bp
rho	KPK_RS25625	F- AACTACGACAAGCCGGAAAA<br>R- ACCGTTACCACG**T**TCCATAC	99 bp
rpoD	KPK_RS03130	F- TCCGGTGCATATGATTGAGA<br>R- ATACGCTCAGCCAGCTCTTC	105 bp

TABLE S27. Primers used on the RT-qPCR analysis of the Fur-regulated genes *cirA*, *iroN* and *fiu* in *K. pneumoniae* strain ATCC 10031. The size of the amplicons are given in base pairs (bp). All primer pairs have the same annealing temperature (60° C).

Gene	Forward primer (5' > 3')	Reverse primer (5' > 3')	Size
cirA	CGACGGCAAGTATGTCCTG	GCTCAGGTTGACCGGATCT	100 bp
iroN	ACCGGGATATTCGCCTGAA	GGCCAGGCTCATTGTAGGT	96 bp
fiu	AGTATGCCGCACTCAACTCC	GGGTACCGCTGTCGGTAGTA	96 bp
